# The Differential Effects of Genetic Mutations in ALS and FTD Genes on Behavioural and Cognitive Changes: A Systematic Review and Meta-Analysis

**DOI:** 10.3390/ijms26136199

**Published:** 2025-06-27

**Authors:** Ana Maria Jiménez-García, Maria Eduarda Tortorella, Agnes Lumi Nishimura, Natalia Arias

**Affiliations:** 1 BRABE Group, Department of Medicine and Health Sciences, Faculty of Life and Natural Sciences, University of Nebrija, C/del Hostal, 28248 Madrid, Spain; 2 Institute Paulo Gontijo, R. Maj. Prado, 42-Indianópolis, São Paulo 04517-020, SP, Brazil; duda9241@terra.com.br (M.E.T.); a.nishimura@qmul.ac.uk (A.L.N.); 3 The Blizard Institute, Faculty of Medicine and Dentistry, Queen Mary University of London, 4 Newark St, London E1 2AT, UK; 4 INEUROPA, Instituto de Neurociencias del Principado de Asturias, Faculty of Psychology, Plaza Feijóo s/n, 33003 Oviedo, Spain; 5 ISPA, Instituto de Investigación Sanitaria del Principado de Asturias, Avenida Hospital Universitario s/n, 33011 Oviedo, Spain

**Keywords:** amyotrophic lateral sclerosis (ALS), frontotemporal dementia (FTD), genetic mutations, behavioural symptoms, cognitive impairment, *C9orf72seq*, *PGRN*, *MAPT*

## Abstract

Amyotrophic lateral sclerosis (ALS) and frontotemporal dementia (FTD) are linked by shared genetic mutations and overlapping clinical features, forming a clinical spectrum. This systematic review and meta-analysis analysed 97 studies, including 3212 patients with key ALS/FTD gene mutations, to identify gene-specific behavioural profiles. *Chromosome 9 open reading frame 72* (*C9orf72*) mutations were strongly associated with psychotic symptoms and aggression, while *superoxide dismutase 1* (*SOD1*) mutations had minimal cognitive effects. *Progranulin* (*PGRN*) mutations correlated with apathy and hallucinations, *microtubule-associated protein tau* (*MAPT*) *mutations* with disinhibition, and *charged multivesicular body protein 2B* (*CHMP2B*) with social impairments. *Fused in sarcoma* (*FUS*) mutations caused early sleep disturbances, *TANK-binding kinase 1* (*TBK1*) led to disinhibition, and *presenilin 1 and 2* (*PSEN1/2*) was linked to severe aggression. Prodromal cognitive changes in *PGRN*, *MAPT*, and *CHMP2B* mutations suggested early disease onset. Despite overlapping symptoms and clinical heterogeneity, understanding gene-specific patterns could inform tailored care strategies to enhance the quality of life for ALS and FTD patients. This study calls for refined guidelines integrating genetic behavioural profiles to improve patient and family support.

## 1. Introduction

Amyotrophic lateral sclerosis (ALS) is a relentlessly progressive neurodegenerative disease characterised primarily by motor neuron degeneration [1], leading to muscle wasting, weakness, and spasticity. Frontotemporal lobar degeneration (FTLD), in contrast, is a neurodegenerative syndrome encompassing a spectrum of neurodegenerative syndromes, including frontotemporal dementia (FTD), semantic dementia (SD), and progressive non-fluent aphasia (PNFA) [2]. The behavioural variant of FTD (bvFTD) is primarily characterised by marked changes in personality, behaviour, and executive dysfunction [3,4]. Semantic dementia (SD) presents with fluent yet empty speech, impaired word comprehension, and a progressive loss of semantic knowledge. Progressive non-fluent aphasia (PNFA) is mainly defined by effortful, non-fluent speech, agrammatism, and motor speech deficits, while single-word comprehension remains relatively preserved in the early stages [5]. Its diagnostic criteria include cognitive impairment, alongside the presence of at least three Rascovsky symptoms, such as disinhibition, apathy or inertia, loss of sympathy or empathy, perseverative or compulsive behaviours, hyperorality, and executive dysfunction [6]. A diagnosis of FTD may also be made in cases where there is a loss of insight and/or the presence of psychotic features accompanied by at least two Rascovsky symptoms, or when language impairment consistent with semantic dementia is present [6]. Moreover, FTD frequently presents with overlapping clinical features of other neurodegenerative conditions, most notably amyotrophic lateral sclerosis (ALS-FTD) or parkinsonism (FTD-P) [7].

Traditionally, ALS was understood as a purely motor disorder. However, accumulating evidence indicates that behavioural and cognitive manifestations are also present in ALS. Neuropsychological screening has demonstrated that 14–40% of ALS patients experience behavioural disturbances, with or without dementia [8]. Approximately 10–15% of ALS patients meet criteria for the behavioural variant of FTD (bvFTD), being diagnosed as ALS-FTD. The remaining ALS patients who do not develop dementia but develop apathy and other behavioural symptoms are classified as ALS with behavioural impairment (ALSbi) [9].

Genetic mutations underlie the pathogenesis of approximately 10% of familial ALS (FALS) and 90% of sporadic ALS (SALS) cases [10]. Mutations in the *microtubule associated protein tau* (*MAPT*), *progranulin* (*PGRN*), *chromosome 9 open reading frame 72* (*C9orf72seq*), *valosin containing protein* (*VCP*) and *charged multivesicular body protein 2B* (*CHMP2B*) are principally associated with FTD. ALS-FTD is mainly correlated with *C9orf72seq* mutations [11]. Transactive response DNA-binding protein (*TARDBP*), *Ubiquilin 2* (*UBQLN2*) and *VCP* mutations are usually linked to ALS. However, although rare, *TARDBP* mutations are also reported in ALS-FTD or FTD cases [12].

Across ALS-FTD and FTD patients, behavioural alterations such as disinhibition, apathy, loss of sympathy/empathy, perseverative or stereotypical behaviour, and dietary alterations are frequently observed [13]. Some features, such as apathy, self-centredness, and irritability, are more prominent in ALS; stereotypies and dietary alterations are more typical in FTD [9]. Despite attempts to distinguish between the behavioural profiles of ALS and FTD, substantial overlap has been observed at clinical, neuropathological, and genetic levels. These findings support the concept of a disease continuum between ALS and FTD [14].

Previous studies have attempted genotype–phenotype correlations for some ALS/FTD-related genes [15,16]. However, substantial gaps remain in our understanding of how specific mutations influence behavioural and cognitive features [17]. Drawing definitive conclusions regarding the differential effects of genetic mutations on patients’ behavioural profiles is challenging, largely due to methodological limitations in the existing studies. These include small sample sizes, variability and inconsistency in assessment tools, and a lack of appropriately matched control groups [10,17]. It is therefore essential to interpret the findings of such studies with caution, particularly as many do not provide quantitative data. Furthermore, identical or distinct mutations within the same gene can result in markedly variable phenotypes, [17] the underlying mechanisms of which remain to be elucidated.

In this systematic review and meta-analysis, we critically examined the differential impact of established ALS- and FTD-related genetic mutations on behavioural and cognitive changes, providing a comprehensive quantitative synthesis of the available evidence.

## 2. Materials and Methods

### 2.1. Search Strategy

A systematic bibliographic search was conducted in accordance with the Preferred Reporting Items for Systematic Reviews and Meta-Analysis (PRISMA) guidelines. Independent searches for original research articles related to genetic mutations in ALS or FTD patients, and healthy patients, and their relationship with cognitive deficits assessed in neuropsychological evaluation were performed in four electronic databases: PubMed, Web of Science (WoS), Science Direct, and Scopus. The search was carried out by two researchers (AJG, NA) on 26 August 2024, using the following terms and search combinations: [“Amyotrophic Lateral Sclerosis”], [“Frontotemporal Dementia OR Frontotemporal Lobar Degeneration”], [“*SOD1*”], [“*FUS*”], [“*C9orf72*”], [“*TARDBP*”], [“*PGRN*”], [“*ATXN2*”], [“*TBK1*”], [“*SQSTM1*”], [“*UBQLN2*”], [“*VCP*”], [“*Tau*”], [“*MAPT*”], [“*CHMP2B*”], [“*OPTN*”], [“*PSEN1*/*2*”], [“*ANXA2*”], [“*DCTN1*”], [“*TIA1*”], [“Mutation”], [“Behavioural OR Behaviour”], [“Neuropsychiatric”], [“Depression OR Depressive”], [“Anxiety OR Anxious”], [“Social Behaviour”], [“Executive Function”], [“Cognitive OR Cognition”], [“Dementia”]. No chronological or methodological filters were applied to the search engines, apart from filtering by titles, keywords, and abstracts. The search strategy was adjusted for all databases. Reference lists of eligible studies were also scanned to include additional publications. All results were compiled into an Excel spreadsheet, and duplicates were discarded.

### 2.2. Literature Selection

Following the removal of duplicates, titles and abstracts of articles retrieved through database searching were screened for eligibility. Studies which did not specifically pertain to the effects of ALS/FTD-related genetic mutations on behavioural changes were deemed ineligible. After the initial screening phase, the full-text articles of selected studies were assessed against the inclusion criteria. For a study to be included in this review, it had to (i) include a study population diagnosed with ALS/FTD according to validated clinical criteria; (ii) report at least one outcome of interest (behavioural changes due to mutations of *SOD1/FUS/C9orf72/TARDBP/PGRN/ATXN2/TBK1/SQSTM1/UBQLN2/VCP/MAPT/Tau/OPTN/TIA1/DCTN1/CHMP2B/PSEN1/PSEN2/ANXA2* genes); (iii) report unique cohorts, and for multiple articles reporting data from the same cohort, the article with the largest sample size was selected to avoid repetition; (iv) be a full-text article with original research; (v) be published in English; and (vi) be published in a peer-reviewed journal.

Exclusion criteria included articles with no abstracts or full texts, editorial publications, systematic reviews, case reports, meta-analyses, and commentaries.

### 2.3. Data Extraction

A total of 20 studies were included in the meta-analysis to evaluate the differential impact of known ALS- and FTD-related genetic mutations on behavioural effects. A continuous random effects model with a standard mean difference was employed to measure the impact of these genetic mutations on behavioural outcomes. Of the 97 studies included in this systematic review, 77 were excluded from the meta-analysis for the following reasons: A total of 39 studies lacked data for the control group, either because a healthy control group was not included, or the controls were not assessed using neuropsychological tests. Additionally, 30 studies were excluded as they were case reports, and 8 studies were excluded due to not reporting the means and standard deviations necessary for the meta-analysis. Statistical significance did not influence the inclusion of these studies in the meta-analysis, and studies reporting null findings were also included.

### 2.4. Statitical Analysis

In the meta-analysis, we evaluated the differential impact of known ALS- and FTD-related genetic mutations on behavioural outcomes. A continuous random effects model was employed using the standard mean difference (SMD) for continuous variables and the odds ratio (OR) for binary variables, both with 95% confidence intervals (CIs).

Statistical heterogeneity between studies was evaluated using the *I*^2^ test. A fixed effect model was applied when the heterogeneity was low (*I*^2^ < 50%, *p* > 0.1), while a random effects model was chosen for higher heterogeneity (*I*^2^ > 50%, *p* < 0.1). Subgroup and meta-regression analyses were conducted to explore potential sources of heterogeneity. For publication bias, Egger’s test was used when the number of included studies was ten or greater.

All data were analysed using R Studio (version 4.3.1) software. The ‘metafor’ package in R was used to calculate the SMD, log odds ratios, CIs, and heterogeneity, while the ‘meta’ package generated the forest plot. Statistical significance was set at *p* < 0.05 for all analyses.

## 3. Results

### 3.1. Study Selection

**Figure 1:**
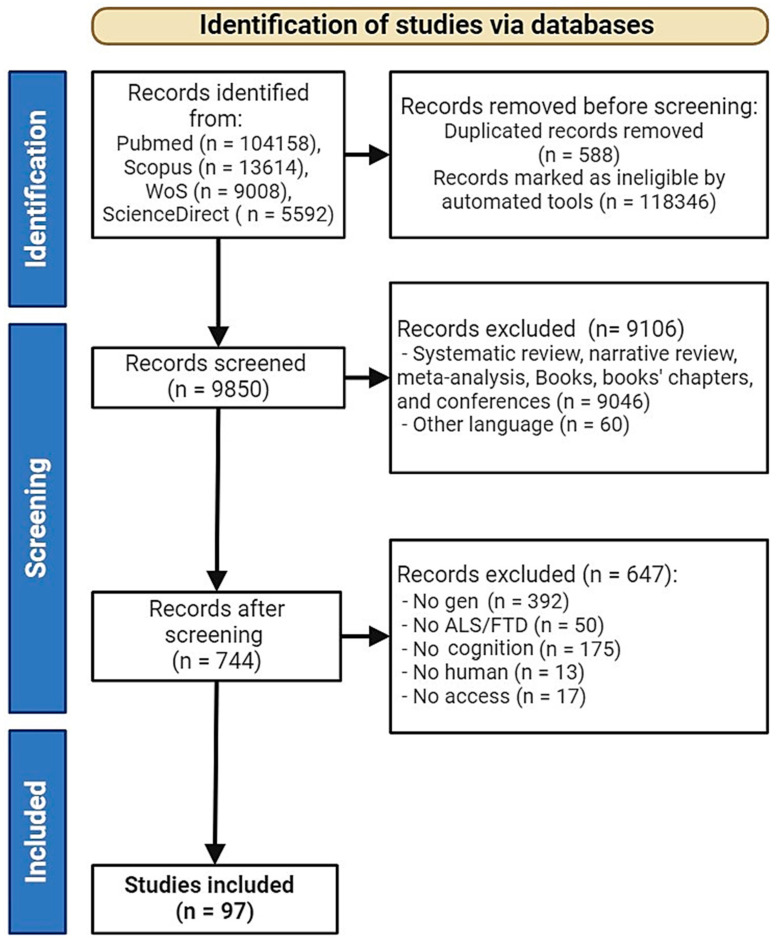
Flowchart of the literature search according to Preferred Reporting Items for Systematic Review and Meta-Analyses (PRISMA). ALS—Amyotrophic Lateral Sclerosis, FTD—Frontotemporal Dementia.

A total of 129,372 studies were identified through searches in WoS, PubMed, SCOPUS, and Science Direct. Initially, 118,346 studies were automatically excluded by the database tools, leaving 10,438 studies. Subsequently, 588 duplicates were removed, reducing the number to 9850. An additional 60 studies were excluded due to language restrictions, resulting in 9790 studies. Following this, 9046 studies were excluded for not being empirical, leaving 744 studies. Finally, after applying additional inclusion and exclusion criteria, a total of 97 studies were included in this systematic review. Studies were excluded due to being outside of the intended date range, missing gene data, and lacking information on cognitive symptoms (see Figure 1).

### 3.2. General Characteristics of Selected Studies

The studies included in this systematic review were published between 2002 and 2024, comprising a mix of cohort and retrospective studies. A total of 97 studies were selected, evaluating the impact of various genetic mutations on cognitive symptoms in patients with frontotemporal dementia (FTD) and amyotrophic lateral sclerosis (ALS). The participants included in these studies had one of the two diagnoses mentioned, commonly either bvFTD or the presence of motor neuron disease (MND).

Across these studies, a total of 3814 patients were studied, encompassing a range of widely researched and novel gene mutations for comparison. The mean age of the participants across these studies was approximately 59 years, aligning with the diverse sample groups analysed. Most studies included database samples of between 200 and 800 participants, while some studies were single-case investigations or had smaller sample sizes, ranging from 10 to 20 participants. Additionally, 30 studies were case reports, including between one and five participants.

The most frequently gene studied was *C9orf72seq*, with 67 studies evaluating 1438 patients. *MAPT* was the focus of 38 studies, involving 906 patients, followed by GRN in 35 studies with 644 individuals. *TARDBP* was studied in eight studies, covering 91 patients, while *FUS* was examined in nine studies involving 18 patients. *SOD1* was evaluated in six studies with 98 patients, and *TBK1* was investigated in five studies with 17 patients. Additionally, a variety of other less common genes linked to FTD, including *PSEN1*, *SQSTM1*, *VCP*, and *ANXA11*, were assessed in smaller patient cohorts across several studies.

A detailed description of all the features is provided in Table 1.

#### 3.2.1. Evaluation of Global Cognition

The results indicate that carriers of *C9orf72seq*, *GRN*, and *MAPT* mutations exhibit a significant global cognitive impairment compared to healthy controls. Notably, *C9orf72seq* mutation carriers demonstrated lower scores on the Mini-Mental State Examination (MMSE) (n = 33), the Montreal Cognitive Assessment (MoCA) (n = 11), the Addenbrooke’s Cognitive Examination—Revised (ACE-R) (n = 1) or ACE-III (n = 2), and the Frontotemporal Dementia Rating Scale (FRS) (n = 3). This cognitive decline is consistently significant when compared to other genetic groups. *GRN* mutation carriers also display a substantial global cognitive decline, with lower MMSE and Frontal Assessment Battery (FAB) scores (n = 4); the Wisconsin Card Sorting Test (WCST) (n = 3) is particularly sensitive to executive dysfunction in these individuals. *MAPT* mutation carriers show lower scores on both MMSE and MoCA, with significant global cognitive impairment, although this is generally less severe than in *GRN* carriers. Commonly used tests for assessing global cognition include the MMSE, MoCA, and Clinical Dementia Rating (CDR) (n = 20). Similar levels of cognitive performance to controls were observed in presymptomatic cases [3,4,25,36,37,44,56,89,96,107].

*TARDBP* patients exhibit a complex profile of cognitive, behavioural, and functional impairments. In terms of global cognition, initial MMSE scores were comparable to healthy controls but declined over time, reflecting the progressive nature of the disease. Memory performance, particularly in immediate recall tasks like the Rey Figure, was notably poor, while other memory functions, such as story recall, showed only minor fluctuations.

Global cognitive decline in *FUS* patients is evident, with MMSE and Addenbrooke’s Mental Test Scores (AMTS) showing a gradual decrease over time, reflecting a worsening cognitive profile. *SOD1* mutation carriers show a trend toward better performance in ALS-specific cognitive scores compared to sporadic ALS and *C9orf72seq* carriers, with statistically significant differences observed only between *SOD1* carriers and sporadic ALS patients.

In patients with *TBK1*-related FTD, global cognitive function initially appeared normal, as evidenced by perfect scores on the MMSE and strong performance on episodic memory tests. However, over time, there was a noticeable decline in cognitive scores, reflecting the progressive nature of the condition.

In individuals carrying the *PSEN1* mutation, a progressive decline in global cognitive functioning was observed, evidenced by a marked reduction in Short Test of Mental Status (STMS) and Mattis Dementia Rating Scale scores, progressing from mild impairment at age 57 to severe dementia by age 59.

Among patients with *SQSTM1* mutations, cognitive impairment was variable. One case exhibited mild cognitive impairment, as indicated by an MMSE score of 27/30, with pronounced short-term memory deficits but relatively preserved long-term memory.

In those with *VCP* mutations, cognitive impairment was present in 25.5% of cases, with frontotemporal dementia (FTD) being the most common presentation. One specific case demonstrated global cognitive decline, with an MMSE score of 25, revealing deficits in orientation, attention, recall, and repetition. Frontal lobe dysfunction was further supported by a score of 12/18 on the Frontal Assessment Battery, indicating impaired executive functions. Other neurological findings were unremarkable; however, mixed cognitive impairments, including FTD, were frequently observed in this population.

Finally, carriers of the *ANXA11* mutation presented with a combined phenotype of ALS and behavioural variant frontotemporal dementia (bvFTD). Cognitive assessment revealed significant impairment, with one patient scoring 20/30 on the MMSE and 15/30 on the MoCA, alongside behavioural symptoms such as irritability and socially inappropriate behaviour. Across patients, considerable variability was observed in cognitive and behavioural symptoms, with MMSE scores ranging from 19 to 27 and MoCA scores from 18 to 25.

#### 3.2.2. Evaluation of Language

In the language domain, the Boston Naming Test (BNT) (n = 9) and category fluency, such as naming animals or words beginning with the letter (e.g., ‘D’) within 1 min (n = 7), are commonly employed, along with other letter fluency assessments. *C9orf72seq* mutation carriers exhibited relatively better performance on naming tests such as the BNT compared to *MAPT* mutation carriers. However, difficulties in verbal fluency and comprehension are evident in both groups, with notably lower scores compared to controls. *GRN* mutation carriers experience more severe deficits in language, including reduced verbal fluency and word retrieval, particularly on the BNT and category fluency tests. *MAPT* mutation carriers also displayed significant language deficits, including poor performance on the BNT and category fluency assessments.

Language abilities, particularly action naming, declined significantly in *TARDBP* mutation carriers, whereas noun naming showed milder but still observable impairments. In contrast, verbal fluency did not differ significantly from that of controls. Similarly, language performance, including tasks such as verbal fluency and the Graded Naming Test, appears comparable between *SOD1* FALS patients and controls, with no notable deficits in action or noun naming. However, patients with the *FUS* mutation demonstrated a marked deterioration, particularly in word recall and phonemic fluency, with evident anomia and semantic errors, although prosopagnosia was not observed. Verbal fluency is notably better preserved in *SOD1* mutation carriers compared to sporadic ALS patients, while no significant differences are found between *C9orf72seq* expansion carriers and sporadic ALS cases.

Language abilities, including naming and vocabulary, progressively declined in *TBK1* mutation carriers, indicating a worsening language impairment over time. A comparable deterioration was observed in *PSEN1* mutation carriers, with naming and verbal fluency impairments evident through performance on the Boston Naming Test and COWAT. In individuals with *SQSTM1* mutations, language was reduced to single-word expressions and was accompanied by mild impairment in verbal fluency.

#### 3.2.3. Evaluation of Visuospatial Skills

Tests commonly used for assessing visuospatial skills include the Rey–Osterrieth Complex Figure Test (n = 3) and the Clock Drawing Test (n = 3). *C9orf72seq* mutation carriers demonstrate difficulties accurately reproducing complex visual information, as seen in the Rey–Osterrieth Complex Figure Test. However, their performance on basic visual perception tasks, assessed by the Visual Object and Space Perception Battery (VOSP), is generally preserved. *GRN* mutation carriers have relatively preserved visuospatial skills in the early stages but develop significant deficits in advanced stages. *MAPT* mutation carriers generally maintain visuospatial abilities, with few significant deficits observed in tasks such as Clock Drawing and visual construction tests. No changes in visuospatial skills were appreciated in *TARDBP* mutation carriers. In contrast, visuoperceptual skills deteriorated significantly in *TBK1* patients, demonstrating severe impairment by the end of the assessment period. Visuoconstructive skills, measured by the Rey–Osterreith Complex Figure test, and visuospatial skills both showed notable declines in *PSEN1* mutation carriers, though line orientation judgement remained intact. Visuospatial difficulties were noted in *SQSTM1* patients, particularly in handling objects and navigating spaces. However, patients with this mutation also exhibited difficulties in visuoconstruction.

#### 3.2.4. Evaluation of Executive Functions

Common tests for assessing executive functions include the Trail Making Test (TMT) (n = 5), the WCST (n = 2), and the Stroop Colour and Word Test (SCWT) (n = 8). Executive functions are significantly affected across all groups, particularly in *C9orf72seq* and *GRN* mutation carriers. *C9orf72seq* carriers show marked deficits in processing speed and verbal fluency, with the SCWT being particularly sensitive to executive dysfunction. *GRN* carriers exhibit difficulties in planning, organisation, and cognitive flexibility, as indicated by the Trail Making Test and the WCST. *MAPT* carriers also display severe executive function deficits, with issues in planning, organisation, and cognitive flexibility detected by the Trail Making Test and the WCST. *TARDBP* subjects’ executive functions remain relatively stable.

Patients with *FUS*-associated FTD exhibit a pronounced executive dysfunction, characterised by difficulties in planning, task shifting, and perseveration. In atypical frontotemporal dementia with ubiquitin inclusions (aFTLD-U), these executive impairments manifest as slowed task initiation, poor engagement, and subcortical–frontal dysfunction, without significant cortical symptoms such as aphasia, agnosia, or spatial disorientation. Conversely, in neuronal intermediate filament inclusion disease (NIFID), memory loss, aphasia, and apraxia are prominent, with more pronounced cortical involvement, leading to potential misdiagnosis as Alzheimer’s disease.

Executive functions, assessed using the Hayling Sentence Completion Test, are comparable between *SOD1* FALS patients and controls. Executive functions are notably better in *SOD1* mutation carriers compared to sporadic ALS patients, with no significant differences between *C9orf72seq* carriers and sporadic ALS patients. Attention and executive functions also exhibited notable declines in *TBK1* mutations carriers, as seen in tasks such as colour–word interference and trail making.

Attention and concentration, assessed through the Trail Making Test and Stroop Test, deteriorated in *PSEN1* mutation carriers, with increased completion times and difficulty handling conflicting information.

Finally, memory, language, and executive functions were variably affected, with measures such as the Trail Making Test (TMT-B), Verbal Fluency Test (VFT), and Stroop Test showing a wide range of performances, indicating differing levels of executive processing speed and cognitive control across the patient cohort.

#### 3.2.5. Evaluation of Memory

For memory assessment, the WAIS-III Digit Span (n = 4) and the California Verbal Learning Test—Short Form (CVLT-SF) (n = 2) are commonly used. *C9orf72seq* carriers experience impairment in episodic and semantic memory, although short-term memory tends to be relatively preserved. *GRN* carriers have significant issues with episodic memory, including immediate recall and word list memory. *MAPT* carriers show substantial deficits in episodic memory, with poor performance on tests such as the CVLT-SF and word recognition.

Memory impairments are also pronounced in patients with *FUS* mutations, particularly affecting episodic recall, although short-term memory remains relatively preserved. In carriers of *TBK1* mutations, memory performance, especially episodic memory, declined significantly, with impairments in word and face recognition becoming apparent after two years. Furthermore, *PSEN1* mutation carriers exhibited severe deficits in memory and learning in both verbal and visual memory tasks.

#### 3.2.6. Evaluation of Attention and Processing Speed

Attention has been assessed using the Trail Making Test (TMT), the Rey Auditory Verbal Learning Test (RAVLT), and the Digit Span. *C9orf72seq* carriers exhibit attention difficulties, evidenced by poorer performance in the Digit Span Backward and sustained attention tasks such as the Modified Trails. *GRN* carriers also show a reduced processing speed and attention, with poor performance on the Number Span Forward test. *MAPT* carriers exhibit deficits in attention and processing speed, with poorer results on tests such as the RAVLT and the Rivermead Behavioural Memory Test (RBMT).

#### 3.2.7. Evaluation of Psychiatric Symptoms

Psychiatric symptoms were evaluated using the Neuropsychiatric Inventory (NPI). In case reports, specific clinical evaluations and psychopathological scales were employed. Perceptual disorders, such as hallucinations and delusions, are more frequent and severe in *C9orf72seq* and FTLD-TDP carriers compared to controls and other genetic groups. *GRN* and *MAPT* carriers exhibit fewer psychotic symptoms but still experience emotional issues such as anxiety and depression. FTLD-TDP patients report high levels of disinhibition and apathy, with notable occurrences of hallucinations. In the *TARDBP* patients, depression and mania were less frequent compared to other groups, but hallucinations and delusions were more prevalent.

#### 3.2.8. Behaviour and Emotion

Emotional issues, such as anxiety and depression, have been commonly assessed using the Hospital Anxiety and Depression Scale (HADS), although other scales like the State–Trait Anxiety Inventory (STAI) or the Beck Depression Inventory have also been used. Clinical behavioural assessments are commonly employed to measure these symptoms. Behavioural changes, including apathy, disinhibition, and repetitive behaviours, are prominent in *C9orf72seq* and FTLD-TDP carriers, alongside emotional symptoms such as anxiety, depression, and hallucinations. *GRN* and *MAPT* carriers also display behavioural changes, though these are generally less severe compared to *C9orf72seq* and FTLD-TDP carriers. In *TARDBP* mutation carriers, behavioural symptoms are marked by high levels of disinhibition, apathy, and hallucinations, with functional abilities in daily living tasks deteriorating progressively, leading to complete dependence. Behaviourally, FUS patients experience increasing disinhibition, impulsivity, and obsessive behaviours, often accompanied by social withdrawal and personality changes characteristic of behavioural variant frontotemporal dementia (bvFTD). High levels of disinhibition (85.7%) and apathy (85.7%) were observed in the FTLD-FUS group, alongside perseveration and hyperorality in 71.4% of cases. Depression is significantly more common in the FTLD-FUS group compared to the FTLD-TDP and FTLD-tau groups, affecting 71.4% of patients, with a lower occurrence of mania and the absence of hallucinations and delusions. As the disease progresses, patients experience worsening dysarthria and dysphagia, a loss of insight, and increasingly compulsive behaviours.

Behavioural measures reveal higher levels of apathy in *SOD1* FALS patients compared to controls, although this is attributed more to the physical limitations of the disease rather than cognitive decline. No significant differences are observed in other behavioural aspects, such as frontal behaviour changes. Emotional lability is elevated in both *SOD1* and sporadic ALS (SALS) patients relative to controls, suggesting that emotional changes can occur independently of cognitive impairment in ALS.

Severe apathy, aggression, and compulsive behaviour were observed in *SQSTM1* mutation carriers. Progressive behavioural changes, including disinhibition, impulsivity, and paranoia, were reported alongside a lack of empathy and emotional expression in other case studies for the same gene.

### 3.3. Meta-Analyses

Of the included papers, a meta-analysis was conducted on cognitive impairment, memory, attention, visuospatial construction, and language. Also, emotional response (depression and anxiety symptoms) and patients’ neurobehavioural and psychiatric symptoms were analysed.

#### 3.3.1. Global Cognition

From the included articles, a meta-analysis was conducted on cognitive impairment, assessed using the Mini-Mental State Examination (MMSE), the Montreal Cognitive Assessment (MoCA), the Clinical Dementia Rating (CDR) plus the National Alzheimer’s Coordinating Center Sum of Boxes (NACC FTLD), the Addenbrooke’s Cognitive Examination (ACE-III), the Frontal Assessment Battery (FAB) and the Frontotemporal Dementia Rating Scale (FRS).

A meta-analysis was conducted using the random effects model to assess cognitive decline, evaluated with the Mini-Mental State Examination (MMSE). A total of 31 studies were included in the analysis. The true outcomes appear to be heterogeneous (*Q*_(30)_ = 319.4069, *p* < 0.0001, *tau*^2^ = 0.5777, *I*^2^ = 92.48%). Significant differences were found in the MMSE (*Z* = −15.0143; *p* < 0.0001). Sub-analyses were performed according to the gene affected in the studies included in the meta-analysis, being significant for *c9orf72seq* (*Z* = −15.5048; *p* < 0.0001), *GRN* (*Z* = −18.1524; *p* < 0.0001), and *MAPT* (*Z* = −5.3780; *p* < 0.0001).

A meta-analysis was conducted to assess cognitive decline, evaluated with the Montreal Cognitive Assessment (MoCA). A total of three studies were included in the analysis. The true outcomes appear not to be heterogeneous (*Q*_(2)_ = 1.4841, *p* = 0.4761, *tau*^2^ = 0.00, *I*^2^ = 0.00%). Significant differences were found in the MoCA (*Z* = −3.5622; *p* = 0.0004).

A meta-analysis was conducted to assess cognitive impairment, evaluated with the Clinical Dementia Rating (CDR) plus the National Alzheimer’s Coordinating Center (NACC FTLD) Sum of Boxes. A total of 22 studies were included in the analysis. The true outcomes appear to be heterogeneous (*Q*_(21)_ = 1696.6578, *p* < 0.0001, *tau*^2^ = 3.0817, *I*^2^ = 98.48%). Significant differences were found in the CDR plus NACC FTLD (*Z* = 11.1972; *p* < 0.0001). Sub-analyses were performed according to the gene affected in the studies included in the meta-analysis, being significant for *c9orf72seq* (*Z* = 8.2216; *p* < 0.0001), GRN (*Z* = 5.8247; *p* < 0.0001), and *MAPT* (*Z* = 4.3178; *p* = 0.0003).

A meta-analysis was conducted to assess the cognitive impairment, evaluated with the Addenbrooke’s Cognitive Examination (ACE-III). A total of five studies were included in the analysis. The true outcomes appear to be heterogeneous (*Q*_(4)_ = 78.5611, *p* < 0.0001, *tau*^2^ = 1.4138, *I*^2^ = 90.05%). Significant differences were found in ACE-III (*Z* = −4.0817; *p* < 0.0001). All the participants who were assessed with the ACE-III had a mutation in the *C9orf72* gene. No other mutations were included in this analysis.

A meta-analysis was conducted to assess cognitive impairment, evaluated with the Frontal Assessment Battery (FAB). A total of three studies were included in the analysis. The true outcomes appear to be heterogeneous (*Q*_(2)_ = 7.6701, *p* = 0.0216, *tau*^2^ = 0.2992, *I*^2^ = 76.14%). Significant differences were found in the FAB (*Z* = −2.7302; *p* = 0.0063).

A meta-analysis was conducted to evaluate cognitive impairment using the Frontotemporal Dementia Rating Scale (FRS). A total of six studies were included in the analysis. The true outcomes appear to be heterogeneous (*Q*_(5)_ = 461.3421, *p* < 0.0001, *tau*^2^ = 3.2109, *I*^2^ = 99.32%). Significant differences in FRS scores were found overall (*Z* = −3.5050; *p* = 0.0005). However, when examining the data by specific genes, no significant differences were observed for *c9orf72seq* (*Z* = −1.7672; *p* = 0.0772), *GRN* (*Z* = −1.4732; *p* = 0.1407), or *MAPT* (*Z* = −1.6227; *p* = 0.1046). This suggests that while there is overall evidence of cognitive impairment, individual genetic factors do not show distinct differences in this analysis.

#### 3.3.2. Memory and Visuospatial Skills

Memory was analysed using the memory subscale of the CBI and the Benson Recall.

A meta-analysis was conducted to assess memory, evaluated with the subscale of the CBI. A total of six studies were included in the analysis. The true outcomes appear to be heterogeneous (*Q*_(22)_ = 63.1966, *p* < 0.0001, *tau*^2^ = 0.5934, *I*^2^ = 92.82%). Significant differences were found in the memory subscale of the CBI (*Z* = 10.5079; *p* < 0.0001). Sub-analyses were performed according to the gene affected in the studies included in the meta-analysis, being significant for *c9orf72seq* (*Z* = 4.5844; *p* < 0.0001), *GRN* (*Z* = 11.2308; *p* < 0.0001), and *MAPT* (*Z* = 7.2684; *p* < 0.0001).

**Figure 2:**
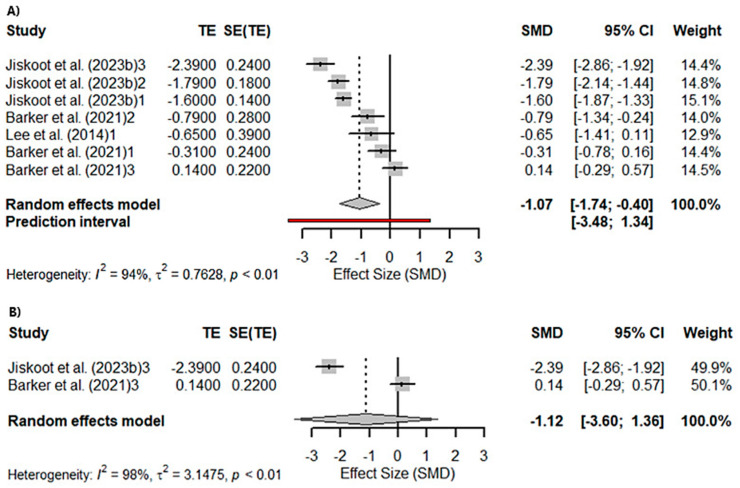
Meta-analysis using a random effects model of selected studies relating to visuospatial memory assessed with the Benson Recall in all patients (*p* = 0.0018) (**A**) and only the MAPT mutation patients (*p* = 0.37) (**B**) [21,56,64]. The plot shows the effect estimates and corresponding confidence intervals (CIs) for each study included in the meta-analysis. The relative weight or contribution of each study to the overall effect estimate is also included in percentages. The overall weighted effect is indicated by a diamond at the bottom of the figure. The labels “1”, “2”, and “3” indicate the genetic groups included in the analysis: 1 = *C9orf72* mutation carriers; 2 = *GRN* mutation carriers; 3 = *MAPT* mutation carriers The figure was generated with *R* software version 4.3.1.

A meta-analysis was conducted to assess visual memory, evaluated with the Benson Recall. A total of five studies were included in the analysis. The true outcomes appear to be heterogeneous (*Q*_(4)_ = 95.9081, *p* = 0.1106, *tau*^2^ = 0.7607, *I*^2^ = 94.08%; Figure 2A). Significant differences were found in the Benson Recall (*Z* = −3.1151; *p* = 0.0018). However, sub-analyses performed according to the gene affected in the studies included in the meta-analysis were significant for *c9orf72seq* (*Z* = −2.1455; *p* = 0.0319) and *GRN* (*Z* = −2.6333; *p* = 0.0085) but not for *MAPT* (*Z* = −0.8878; *p* = 0.3747; Figure 2B).

A meta-analysis was conducted to assess visuoconstructive skills, evaluated with Benson Copy. A total of four studies were included in the analysis. The true outcomes appear not to be heterogeneous (*Q*_(3)_ = 4.0585, *p* = 0.2552, *tau*^2^ = 0.0000, *I*^2^ = 0.01%). Significant differences were found in Benson Copy (*Z* = −13.1915; *p* < 0.0001). Similarly, sub-analyses performed according to the gene affected in the studies included in the meta-analysis were significant for *c9orf72seq* (*Z* = −2.7351; *p* = 0.0062).

#### 3.3.3. Attention

Attention was analysed using the Digit Spam (DS) Forward and Backward, and the Trail Making Test Part A (TMT-A) and B (TMT-B).

A meta-analysis was conducted to assess attention, evaluated with DS Forward. A total of four studies were included in the analysis. The true outcomes appear not to be heterogeneous (*Q*_(3)_ = 3.8627, *p* = 0.2767, *tau*^2^ = 0.0222, *I*^2^ = 17.40%). No significant differences were found in DS Forward (*Z* = −1.2189; *p* = 0.2229). Sub-analyses were performed according to the gene affected in the studies included in the meta-analysis, being not significant for *c9orf72seq* (*Z* = −1.6057; *p* = 0.1083) or *MAPT* (*Z* = −0.0587; *p* = 0.9532).

A meta-analysis was conducted to assess attention, evaluated with DS Backward. A total of seven studies were included in the analysis. The true outcomes appear to be highly heterogeneous (*Q*_(6)_ = 47.0155, *p* < 0.0001, *tau^2^* = 8.6971, *I*^2^ = 98.74%). No significant differences were found in DS Backward (*Z* = 0.6606; *p* = 0.5089). Sub-analyses were performed according to the gene affected in the studies included in the meta-analysis, being not significant for *c9orf72seq* (*Z* = 0.7360; *p* = 0.4617) or *MAPT* (*Z* = 0.5211; *p* = 0.6023).

A meta-analysis was conducted to assess sustained attention, evaluated with TMT-A. A total of three studies were included in the analysis (*k* = 6). The true outcomes appear to be moderate heterogeneous (*Q*_(2)_ = 7.8111, *p* = 0.0201, *tau*^2^ = 0.1667, *I*^2^ = 72.86%). Significant differences were found in TMT-A (*Z* = 2.2225; *p* = 0.0262).

A meta-analysis was conducted to assess executive functioning, evaluated with TMT-B. A total of three studies were included in the analysis (k = 3). The true outcomes appear to be heterogeneous (*Q*_(2)_ = 9.7358, *p* = 0.0077, *tau*^2^ = 0.2430, *I*^2^ = 79.64%). Significant differences were found in TMT-B (*Z* = 2.7108; *p* = 0.0067).

#### 3.3.4. Language

Language was analysed using the Boston Naming Test (BNT) to measure confrontational word retrieval. On the other hand, semantic impairment was assessed by the Camel and Cactus Test and by requesting the writing of as many animals as they could think of for 1 min.

A meta-analysis was conducted to assess naming, measured with the BNT. A total of four studies were included in the analysis. The true outcomes appear to be highly heterogeneous (*Q*_(3)_ = 23.1580, *p* < 0.0001, *tau*^2^ = 1.0237, *I*^2^ = 89.34%). Significant differences were found in BNT (*Z* = −3.7154; *p* = 0.0002). Sub-analyses performed according to the gene affected in the studies included in the meta-analysis were significant for *c9orf72seq* (*Z* = −7.0829; *p* < 0.0001).

**Figure 3:**
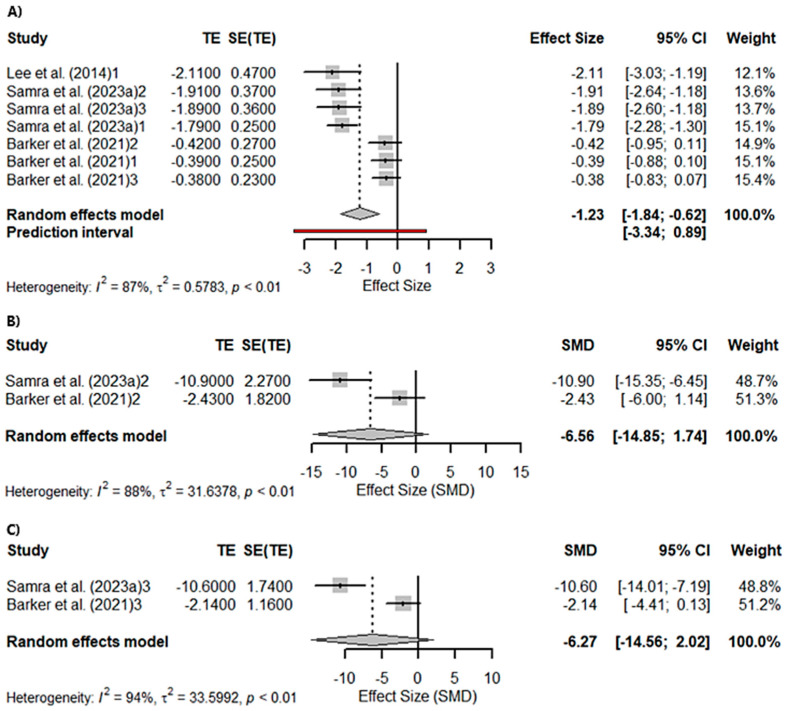
Meta-analysis using a random effects model of selected studies relating to semantic fluency assessed with the “animals in 1 min” in all patients (*p* < 0.0001) (**A**), only the GRN mutation patients (*p* = 0.13) (**B**), and only the MAPT mutation patients (*p* = 0.14) (**C**) [21,64,82]. The plot shows the effect estimates and corresponding confidence intervals (CIs) for each study included in the meta-analysis. The relative weight or contribution of each study to the overall effect estimate is also included in percentages. The overall weighted effect is indicated by a diamond at the bottom of the figure. The labels “1”, “2”, and “3” indicate the genetic groups included in the analysis: 1 = *C9orf72* mutation carriers; 2 = *GRN* mutation carriers; 3 = *MAPT* mutation carriers. The figure was generated with *R* software version 4.3.1.

A meta-analysis was conducted to assess language, evaluated through semantic frequency (animals in 1 min). A total of seven studies were included in the analysis. The true outcomes appear to be heterogeneous (*Q*_(4)_ = 46.6999, *p* < 0.0001, *tau*^2^ = 0.5801, *I*^2^ = 87.47%). Significant differences were found in semantic fluency (*Z* = −3.9284; *p* < 0.0001). Sub-analyses performed according to the gene affected in the studies included in the meta-analysis were significant for *c9orf72seq* (*Z* = −2.6964; *p* = 0.0070) but not for *GRN* (*Z* = −1.5330; *p* = 0.1253) or *MAPT* (*Z* = −1.4691; *p* = 0.1418). On the other hand, a total of six studies were included in the analysis of semantic impairment assessed with the Camel and Cactus Test. The true outcomes appear to be highly heterogeneous (*Q*_(5)_ = 66.7411, *p* < 0.0001, *tau*^2^ = 0.4644, *I*^2^ = 90.00%). Significant differences were found in semantic impairment (Z = −7.4356; *p* < 0.0001; Figure 3A). Similarly, sub-analyses performed according to the gene affected in the studies included in the meta-analysis were significant for *c9orf72seq* (*Z* = −3.7858; *p* = 0.0002), *GRN* (*Z* = −7.2035; *p* < 0.0001; Figure 3B), and *MAPT* (*Z* = −7.6944; *p* < 0.0001; Figure 3C).

#### 3.3.5. Emotion

On the other hand, the emotional response of the patients was analysed using the Hospital Anxiety and Depression Scale (HADS).

A meta-analysis was conducted to assess depression, measured with the HADS. A total of three studies were included in the analysis. The true outcomes appear not to be heterogeneous (*Q*_(2)_ = 0.7812, *p* = 0.6766, tau^2^ = 0, *I*^2^ = 0.00%). Significant differences were found in depression (*Z* = 3.0057; *p* = 0.0027). Sub-analyses were performed according to the gene affected in the studies included in the meta-analysis, which was significant for *SOD*-1 (*Z* = 2.8566; *p* = 0.0043).

A meta-analysis was conducted to assess anxiety, measured with the HADS. A total of three studies were included in the analysis. The true outcomes appear not to be heterogeneous (*Q*_(2)_ = 3.1005, *p* = 0.2122, *tau*^2^ = 0.0445, *I*^2^ = 31.45%). Significant differences were found in anxiety (*Z* = 0.7236; *p* = 0.4693). Sub-analyses were performed according to the gene affected in the studies included in the meta-analysis, which was not significant for *SOD*-1 (*Z* = 0.8113; *p* = 0.4172).

#### 3.3.6. Neurobehavioural and Psychiatric Symptoms

Patients’ neurobehavioral and psychiatric symptoms were also analysed, including daily living skills, self-care skills, mood changes, bizarre beliefs, eating habits, abnormal behaviours, sleep, stereotyped motor behaviours, and reduced motivation from the subscales of the Cambridge Behavioural Inventory Revised (CBI).

A total of six studies were included in the analysis of everyday skills. The true outcomes appear to be heterogeneous (*Q*_(5)_ = 30.6372, *p* < 0.0001, *tau*^2^ = 0.2080, *I*^2^ = 83.41%). Significant differences were found in everyday skills (*Z* = 12.8595; *p* < 0.0001). Sub-analyses were performed according to the gene affected in the studies included in the meta-analysis, being significant for *c9orf72seq* (*Z* = 5.1538; *p* < 0.0001), *GRN* (*Z* = 13.7429; *p* < 0.0001), and *MAPT* (*Z* = 5.8062; *p* < 0.0001).

A total of six studies were included in the analysis of self-care skills. The true outcomes appear not to be heterogeneous (*Q*_(5)_ = 2.9616, *p* = 0.7059, tau^2^ = 0, *I^2^* = 0.00%). Significant differences were found in self-care skills (*Z* = 10.8165; *p* < 0.0001). Sub-analyses were performed according to the gene affected in the studies included in the meta-analysis, being significant for *c9orf72seq* (*Z* = 7.9004; *p* < 0.0001), *GRN* (*Z* = 5.4006; *p* = 0.0043), and *MAPT* (*Z* = 5.3070; *p* = 0.0001).

A total of six studies were included in the analysis of mood changes. The true outcomes appear not to be heterogeneous (*Q*_(5)_ = 5.0131, *p* = 0.4143, *tau*^2^ = 0.0021, *I*^2^ = 6.20%). Significant differences were found in mood changes (*Z* = 17.7143; *p* < 0.0001). Sub-analyses were performed according to the gene affected in the studies included in the meta-analysis, being significant for *c9orf72seq* (*Z* = 12.2727; *p* < 0.0001), *GRN* (*Z* = 6.6881; *p* < 0.0001), and *MAPT* (*Z* = 9.2339; *p* < 0.0001).

A total of six studies were included in the analysis of odd beliefs. The true outcomes appear not to be heterogeneous (*Q*_(5)_ = 1.7272, *p* = 0.8855, *tau*^2^ = 0, *I*^2^ = 0.00%). Significant differences were found in odd beliefs (*Z* = 7.9739; *p* < 0.0001). Sub-analyses were performed according to the gene affected in the studies included in the meta-analysis, being significant for *c9orf72seq* (*Z* = 5.0640; *p* < 0.0001), *GRN* (*Z* = 2.8566; *p* = 0.0043), and *MAPT* (*Z* = 3.7976; *p* = 0.0001).

A total of six studies were included in the analysis of eating habits. The true outcomes appear to be heterogeneous (*Q*_(5)_ = 48.7983, *p* < 0.0001, tau^2^ = 0.4332, *I*^2^ = 91.59%). Significant differences were found in eating habits (*Z* = 9.1995; *p* < 0.0001). Sub-analyses were performed according to the gene affected in the studies included in the meta-analysis, being significant for *c9orf72seq* (*Z* = 5.7407; *p* < 0.0001), *GRN* (*Z* = 6.1056; *p* < 0.0001), and *MAPT* (*Z* = 6.1907; *p* < 0.0001).

A total of six studies were included in the analysis of abnormal behaviour. The true outcomes appear to be heterogeneous (*Q*_(5)_ = 91.1562, *p* < 0.0001, *tau*^2^ = 0.3120, *I*^2^ = 93.19%). Significant differences were found in abnormal behaviour (*Z* = 10.1224; *p* < 0.0001). Sub-analyses were performed according to the gene affected in the studies included in the meta-analysis, being significant for *c9orf72seq* (*Z* = −7.8000; *p* < 0.0001), *GRN* (*Z* = 6.3129; *p* < 0.0001), and *MAPT* (*Z* = 16.8402; *p* < 0.0001).

A total of six studies were included in the analysis of sleep. The true outcomes appear not to be heterogeneous (*Q*_(5)_ = 2.5827, *p* = 0.7640, *tau*^2^ = 0, *I*^2^ = 0.00%). Significant differences were found in sleep (*Z* = 9.1693; *p* < 0.0001). Sub-analyses were performed according to the gene affected in the studies included in the meta-analysis, being significant for *c9orf72seq* (*Z* = 6.2292; *p* < 0.0001), *GRN* (*Z* = 5.5088; *p* = 0.0043), and *MAPT* (*Z* = 3.0225; *p* = 0.0025).

A total of six studies were included in the analysis of stereotypic and motor behaviours. The true outcomes appear to be heterogeneous (*Q*_(5)_ = 14.9692, *p* = 0.0105, tau^2^ = 0.4321, *I*^2^ = 68.21%). Significant differences were found in stereotypic and motor behaviours (*Z* = 8.1324; *p* < 0.0001). Sub-analyses were performed according to the gene affected in the studies included in the meta-analysis, being significant for *c9orf72seq* (*Z* = 3.6949; *p* < 0.0001), *GRN* (*Z* = 6.3292; *p* < 0.0001), and *MAPT* (*Z* = 3.0101; *p* = 0.0026).

A total of six studies were included in the analysis of reduced motivation. The true outcomes appear to be heterogeneous (*Q*_(5)_ = 17.4170, *p* = 0.0038, *tau*^2^ = 0.5156, *I*^2^ = 71.67%). Significant differences were found in reduced motivation (*Z* = 7.6225; *p* < 0.0001). Sub-analyses were performed according to the gene affected in the studies included in the meta-analysis, being significant for *c9orf72seq* (*Z* = 8.6801; *p* < 0.0001), *GRN* (*Z* = 3.7614; *p* = 0.0002), and *MAPT* (*Z* = 7.4340; *p* < 0.0001).

## 4. Discussion

This systematic review and meta-analysis evaluate the complex and distinct behavioural profiles associated with different genetic mutations within the ALS-FTD spectrum. By examining cognitive and behavioural outcomes in relation to specific gene mutations, significant variability was observed, reinforcing the notion that genetic subtypes within ALS and FTD are characterised by unique clinical presentations. These findings underscore the importance of considering genetic underpinnings when evaluating and managing patients within this clinical continuum. The results are discussed by gene, highlighting the salient behavioural and cognitive features identified.

### 4.1. C9orf72seq

Patients with the *C9orf72seq* repeat expansion consistently exhibited the most pronounced cognitive and behavioural impairments across multiple domains. In terms of global cognition, these patients showed significantly greater deterioration domains compared to those with *GRN* and *MAPT* mutations, as evidenced by lower scores on the MMSE, CDR-FTD-Sum of Boxes, and the FRS. Although memory was not the most severely impaired domain, *C9orf72seq* carriers still demonstrated notable deficits, particularly in specific recall tasks [32,64,65,79]. Attention was also significantly affected, with substantial difficulties observed on the TMT, indicating considerable attentional dysfunction. However, the DS Backward and Forward tests failed to detect significant differences, suggesting that attentional deficits in *C9orf72seq* patients may be more nuanced and dependent on task complexity [64].

In the language domain, *C9orf72* repeat expansion carriers were the most severely affected, consistently demonstrating poor performance on the BNT, semantic fluency tasks, and the Camel and Cactus Test, indicating a broad and pervasive language impairment [64]. While semantic fluency assessments, such as generating animal names in one minute, did not differentiate *GRN* and *MAPT* mutation carriers from healthy controls, they clearly identified deficits in *C9orf72seq* patients [82].

With respect to neurobehavioural and psychiatric symptomatology, *C9orf72seq* mutation carriers again stood out, particularly in areas such as self-care abilities, mood dysregulation, sleep disturbances, unusual beliefs, and diminished motivation. This constellation of symptoms suggests a unique behavioural phenotype characterised by cognitive deterioration, pronounced language dysfunction, and severe psychiatric features, collectively resulting in substantial impairments in everyday functioning and quality of life [48,73].

The *C9orf72seq* repeat expansion is associated with widespread and severe neurodegeneration, particularly affecting the frontal and temporal lobes. This mutation leads to significant disruptions in global cognitive performance, with patients exhibiting pronounced deficits across multiple cognitive domains, including memory, attention, and language. The profound impact on global cognition is likely due to extensive neuroanatomical involvement, particularly within the prefrontal cortex, which is crucial for executive function, working memory, and attention.

The marked language difficulties observed in tasks like the BNT and semantic fluency may be linked to degeneration in the left perisylvian language network, including Broca’s area and temporal regions involved in semantic processing. The severe attention deficits detected on the TMT, but not in simpler tasks like DS Backward and Forward, suggest that the *C9orf72seq*-related pathology may disproportionately affect more complex, multitasking cognitive functions, possibly due to prefrontal cortical degeneration.

The pronounced neurobehavioural and psychiatric symptoms, such as mood changes, sleep difficulties, and reduced motivation, likely reflect the widespread involvement of the limbic system and other subcortical structures, which are critical for emotional regulation and motivation. The broad spectrum of impairments in *C9orf72seq* carriers underscores the extensive and multifaceted impact of this mutation on both cognitive and behavioural functions.

### 4.2. Granulin

Patients with *GRN* mutations exhibited a distinct profile of cognitive and behavioural impairments, generally less severe than those observed in *C9orf72* expansion carriers. In terms of global cognition, *GRN* mutation carriers demonstrated moderate impairment, mirroring the decline seen in *C9orf72* patients, albeit to a lesser extent. The most striking finding in this group was in the domain of memory, with *GRN* patients exhibiting the most pronounced impairments, as reflected in a poor performance on both the CBI and Benson Recall task [21,33]. The Benson Recall task appeared particularly sensitive to memory impairment in *GRN* mutation carriers yet failed to identify similar deficits in *MAPT* patients, highlighting the specificity of the memory dysfunction associated with *GRN’s* pathology.

With respect to attention, *GRN* patients did not show the same level of impairment as *C9orf72seq* patients, and, consistent with other groups, performance on DS (Backwards and Forwards) was of limited diagnostic value. Language abilities in *GRN* patients were also comparatively preserved, with no significant differences observed in semantic fluency tasks relative to healthy controls. These findings suggest that while GRN mutations do affect language, the impact is relatively mild and more selective than that observed in C9orf72 mutation carriers [83,102].

In terms of neurobehavioural and psychiatric symptoms, *GRN* patients displayed significant impairments, particularly in everyday skills, stereotypic motor behaviours, and reduced motivation. These findings indicate that while *GRN* carriers experience considerable behavioural difficulties, these are distinct from those observed in *C9orf72seq* carriers, with a more pronounced impact on routine activities and motor functions [83].

Overall, patients with *GRN* mutations present a cognitive–behavioural profile characterised by severe memory impairment, alongside moderate difficulties in other domains. The significant memory deficits, particularly in tasks like the CBI and Benson Recall, suggest that *GRN* mutations may lead to pronounced hippocampal and parietal lobe atrophy, regions crucial for memory consolidation and spatial navigation.

The relatively preserved global cognition and language functions in *GRN* patients could be due to a more selective neurodegeneration, sparing the frontal and temporal regions to a greater extent than in *C9orf72seq* mutation carriers. The moderate impact on attention, with less severe impairment than seen in *C9orf72seq* carriers, also supports the idea of more focal neurodegenerative processes in *GRN*-related pathology.

Patients with *GRN* mutations demonstrate impairments in everyday functioning, stereotyped motor behaviours, and apathy, features associated with dysfunction in the frontoparietal networks essential for motor planning and goal-directed activity. Degeneration in the parietal lobe disrupts sensory integration and visuospatial coordination, thereby impairing the execution of daily tasks. Concurrently, involvement of the frontal lobe, critical for executive planning and inhibitory control, accounts for the presence of repetitive behaviours and a diminished ability to adjust motor responses, alongside reduced motivation or apathy.

In contrast to *C9orf72seq* expansion carriers, who typically present with more widespread cognitive impairment across multiple domains, carriers of *GRN* mutations display a more circumscribed pattern of neurodegeneration, with certain cognitive functions, such as language and aspects of memory, relatively preserved. This selective frontoparietal involvement highlights the importance of network-based brain organisation in mediating the cognitive and behavioural phenotypes observed in neurodegenerative conditions.

### 4.3. MAPT

The behavioural and cognitive profile of patients with *MAPT* mutations differed considerably from those with *C9orf72seq* and *GRN* mutations. In terms of global cognition, *MAPT* patients were the least impaired among the three groups, with relatively preserved scores across the MMSE, CDR-FTD-Sum of Boxes, and FRS [21]. Notably, memory was also less affected in MAPT carriers, as evidenced by the Benson Recall task, which failed to detect significant deficits in this group—sharply contrasting with the findings in *GRN* mutation carriers [96].

Regarding attention, *MAPT* mutation carriers did not exhibit the same level of impairment seen in *C9orf72seq* carriers, and the DS Backward and Forward tasks again were not sensitive enough to highlight any differences compared to controls. Language abilities in *MAPT* patients were similarly preserved, with semantic fluency tasks, including the generation of animal names, failing to distinguish *MAPT* patients from controls [82]. This suggests that while cognitive and behavioural changes do occur in *MAPT* carriers, they are less severe and more specific compared to the other groups.

In the assessment of neurobehavioural and psychiatric symptoms, *MAPT* mutation carriers were most impaired in abnormal behaviours, which distinguished them from the other groups. Although they showed significant differences from controls, the impairment profile was distinct, with less impact on daily skills and motivation but a greater presence of behavioural abnormalities, aligning with the recognised phenotype associated with *MAPT* mutations [36].

Overall, the *MAPT* mutation is associated with the least severe cognitive impairment among the three genetic subtypes, with global cognitive functioning largely preserved and notable deficits confined to specific areas, such as abnormal behaviour. The relative preservation of memory functions, as evidenced by performance on the Benson Recall task, suggests that *MAPT*-related pathology may primarily affect the anterior temporal and frontal lobes, with limited involvement of the hippocampal structures critical for memory consolidation.

The absence of marked deficits in attention and language tasks, particularly in tasks like DS (Backwards and Forwards) and semantic fluency, may reflect the predominant localisation of tau pathology within the frontal and temporal cortices, sparing regions more directly implicated in basic attentional processes and core language functions. The selective disruption in behavioural domains points towards a focal impact of *MAPT* mutations on neural circuits subserving social cognition and behavioural regulation, notably within the orbitofrontal cortex and anterior cingulate gyrus.

The relatively preserved cognitive profile in *MAPT* mutation carriers suggests that tau pathology may initially affect brain regions involved in behavioural regulation and social cognition, with less immediate impact on core cognitive functions. This could explain why *MAPT* mutation carriers do not exhibit the same breadth of cognitive impairments seen in *C9orf72seq* or *GRN* mutations.

### 4.4. TARDBP

Patients carrying mutations in the *TARDBP* gene, which has been associated with both ALS and FTD, frequently exhibit early deterioration in executive function [3,26,34,67,84,95,110]. This is most notably manifested as difficulties in planning, cognitive flexibility, and perseveration—hallmark features of subcortical–frontal dysfunction. Affected individuals may appear disengaged and demonstrate reduced initiative, often observed as vacant staring or an inability to complete tasks. Memory performance in *TARDBP* mutation carriers is often affected indirectly, with inefficiencies that align with the executive impairments, but overall memory remains intact relative to the profound frontal dysfunction [26,95]. As the disease progresses, these patients may exhibit behavioural disinhibition, impulsivity, and obsessive–compulsive tendencies, leading to the significant personality changes that characterise bvFTD. Hallucinations, apathy, and compulsive behaviours are common, and motor symptoms associated with ALS can emerge as well, adding to the complexity of their condition [26].

### 4.5. FUS

The *FUS* mutation is characterised by executive function deficits and subcortical–frontal dysfunction. These patients particularly struggle with planning and task shifting, often becoming stuck on repetitive actions or thought patterns. Interestingly, *FUS*-related FTD patients do not typically show early cortical symptoms like aphasia or apraxia, which can make the presentation more subtle initially compared to other forms of FTD [29]. Memory impairments in *FUS*-related cases are generally secondary to executive dysfunction rather than primary memory loss [29,51]. Over time, however, patients exhibit behavioural changes such as disinhibition and impulsivity, leading to social withdrawal, loss of decorum, and obsessive behaviours [50]. Depression is highly prevalent in these patients, alongside hyperorality and compulsive behaviour. *FUS* mutation carriers show a particularly high degree of disinhibition and emotional dysregulation, often leading to more severe behavioural disturbances than in other forms of FTD [110].

### 4.6. SOD1

Patients with *SOD1* mutations display a cognitive profile that is relatively preserved compared to other ALS-linked genes [67,101,106]. Many *SOD1* mutation carriers perform similarly to healthy controls on cognitive tasks, showing no significant memory impairment, language deficits, or executive dysfunction. However, *SOD1* mutation carriers exhibit a distinct emotional profile, with higher levels of emotional lability, suggesting that while cognitive functions remain intact, emotional regulation is significantly affected [105,106]. Apathy is also more common in these patients, but it is thought to be related to physical limitations rather than intrinsic cognitive decline [67].

### 4.7. PSEN1, SQSTM1, VCP, and ANXA11

The remaining ALS/FTD cases with mutations in different genes, as presented in Table 1 (e.g., *PSEN1*, *Tau*, *VAPB*, *TBK1*, *KIF5A*, etc.), did not have a sufficient sample size for a comprehensive comparison. This limitation hindered our ability to draw reliable conclusions. Mutations in these genes are rare, and this study was constrained by the challenge of obtaining an adequate number of cases for a meta-analysis. However, it is important to mention that the results from patients with *PSEN1*, *SQSTM1*, *VCP*, and *ANXA11* mutations highlight distinct patterns of cognitive impairment, reflecting both shared and gene-specific features of neurodegeneration. All these mutations are linked to varying degrees of cognitive impairment, though the onset and severity differ. In *PSEN1* mutation carriers, a rapid and severe cognitive decline is prominent, as shown by the significant drop from mild to severe dementia within a two-year period. This reflects the aggressive nature of *PSEN1*-associated familial Alzheimer’s disease (AD), known for its early onset and rapid progression [98]. In contrast, *SQSTM1* and *ANXA11* mutations present with a milder initial impairment but progressively worsen, showing a variability in cognitive symptoms even in the early stages of FTD associated with these mutations [61].

Interestingly, patients with *VCP* mutations also exhibit a substantial prevalence of cognitive impairment; however, the predominance of FTD as the primary cognitive phenotype suggests that these patients may initially present with a more focal executive dysfunction, in contrast to the more global cognitive decline observed in *PSEN1* mutation carriers [87].

Memory impairment is a key feature in *PSEN1* mutation carriers, with both verbal and visual memory severely affected. The memory deficits in *SQSTM1* and *ANXA11* mutations are less pronounced early on, but *SQSTM1* cases still show short-term memory loss, while long-term memory remains intact [61]. In *ANXA11* mutation carriers, the range of memory deficits is broader, with scores on memory tasks showing variability [104]. These findings suggest that while *PSEN1* mutations follow a more typical Alzheimer’s-like memory impairment, *SQSTM1* and *ANXA11* patients may experience more selective or progressive memory deficits that are reflective of FTD’s pathology.

Executive dysfunction is a hallmark feature across all mutations, though the degree and nature vary. *PSEN1* carriers show marked impairments in planning, problem-solving, and cognitive flexibility. This highlights the early involvement of the frontal lobes in *PSEN1*-related dementia, despite its strong Alzheimer’s phenotype. *SQSTM1*, *VCP*, and *ANXA11* mutations, linked to FTD, also show substantial executive dysfunction, with patients displaying deficits in attention, task switching, and inhibition control. Also, the *VCP*-related FTD results supported the impact of the mutation on executive function at early stages.

Regarding language impairments, *PSEN1* carriers show significant difficulty with naming, verbal fluency, and language retrieval. In *SQSTM1*, *VCP,* and *ANXA11* mutations, language impairment appears more variable, with some patients exhibiting speech apraxia or grammatical issues [27,61,98]. This reflects the heterogeneity of language deficits in FTD, which may range from non-fluent aphasia to more semantic language impairments, depending on the specific mutation and its effects on the temporal and frontal lobes.

Visuospatial skills are notably impaired in *PSEN1* mutation carriers [98]. In contrast, *SQSTM1*, *VCP*, and *ANXA11* mutations show a more mixed pattern of visuospatial decline. For example, *VCP* mutation carriers often have preserved visuospatial skills early on, but visuoconstructional abilities may deteriorate as the disease progresses [87,111]. The variability in visuospatial decline across FTD-related mutations indicates that while some patients experience early impairments, others may retain these abilities until later stages.

Finally, behavioural disturbances are a common feature across *SQSTM1*, *VCP*, and *ANXA11* mutations, all of which are linked to FTD syndromes. *SQSTM1* patients, in particular, showed severe apathy, disinhibition, and aggressive behaviour, alongside compulsive tendencies [61]. Similarly, *VCP* mutation carriers often develop behavioural variant FTD (bvFTD), characterised by social withdrawal, irritability, and compulsive behaviours [3,50]. These behavioural features are less prominent in *PSEN1* mutation carriers, where personality changes tend to occur later in the disease’s course, likely due to the primarily cognitive nature of AD [98]. The presence of significant emotional and behavioural changes in FTD-associated mutations underscores the involvement of the frontal lobes, particularly in social cognition and impulse control.

### 4.8. Limitations and Future Directions

While our findings indicate that specific behavioural impairments may be significantly increased in certain subgroups of ALS or FTD patients, we also acknowledge several limitations of the present meta-analysis. A major limitation lies in the inability to evaluate certain genes due to a lack of studies meeting the inclusion criteria. This highlights an uneven distribution of research attention across the genetic mutations implicated in ALS and FTD, potentially biasing our understanding of the broader genetic and behavioural spectrum of these disorders.

While *C9orf72seq*, *GRN*, and *MAPT* mutations have been extensively associated with behavioural dysregulation, other genes such as *CHMP2B*, *FUS*, and *TBK1* remain under-investigated. While individuals with mutations in these genes often present with cognitive and behavioural deficits resembling FTD phenotypes, the limited number of patients with these mutations and the lack of research on less commonly studied mutations hinder comprehensive meta-analytic comparisons. This hampers our understanding of the behavioural and cognitive phenotypes associated with these mutations. Additionally, the lack of robust data may contribute to diagnostic bias, where certain genetic mutations are under-recognised or misinterpreted due to the absence of well-established behavioural and cognitive markers.

This research gap highlights a critical need for more comprehensive investigations into these underrepresented genetic mutations. Future studies should incorporate well-matched control groups and standardised behavioural and cognitive assessment tools to provide a more holistic understanding of the ALS-FTD spectrum. Such an expansion of the research focus would facilitate more accurate diagnoses and the development of tailored therapeutic strategies.

Another limitation of this meta-analysis is the relative paucity of studies that include healthy control groups, thereby limiting the scope of cross-sectional comparisons across behavioural and cognitive domains. This shortcoming reinforces the necessity for future investigations to include appropriately matched controls to enable a more accurate delineation of the specific effects of genetic mutations on neuropsychological functioning. The observed clinical heterogeneity within identical mutations, along with phenotypic overlap between different genetic variants, further complicates the identification of distinct behavioural profiles. These challenges underscore the importance of employing refined assessment tools tailored to genetic subtypes within the ALS–FTD continuum.

Additionally, a significant challenge encountered in this meta-analysis was the limited ability to evaluate tests like the ECAS (Edinburgh Cognitive and Behavioural ALS Screen), which are specifically designed for ALS and FTD patients. The ECAS is essential in clinical settings for detecting cognitive and behavioural changes in individuals with ALS. However, its utility in broader comparative analyses is constrained by the fact that such tests are rarely, if ever, administered to healthy control groups. This limitation presents a significant barrier to meta-analytic comparisons, as meta-analyses rely on synthesising data across studies to establish generalisable conclusions about the effectiveness or diagnostic utility of various tests. Without control group data, it becomes impossible to determine the specificity and sensitivity of tests like the ECAS relative to the general population, limiting our ability to compare the cognitive profiles of ALS/FTD patients with those of unaffected individuals and to establish normative data.

The lack of normative data for the ECAS further limits the interpretability of its outcomes. Although the ECAS is invaluable for monitoring cognitive decline in ALS/FTD, without data from non-affected individuals, it remains challenging to determine whether the detected impairments are specific to these conditions or may reflect broader neurodegenerative processes or even normal ageing. This gap in the literature underscores the need for studies that administer the ECAS or similar ALS/FTD-specific tools to healthy controls. Such data would allow researchers to refine their understanding of these tests’ discriminatory power and place within the broader context of neuropsychological assessment.

## 5. Conclusions

In conclusion, this systematic review and meta-analysis have identified that specific behavioural outcomes are frequently observed in subtypes of ALS/FTD cases, segregated by genes. Validated diagnostic investigations and biomarkers, as well as confounding phenotypes with other conditions, may delay the diagnosis of ALS and, thus, the treatment. Given the link between genetic mutations and behavioural outcomes, refining patient care guidelines to consider specific behavioural characterisations based on the underlying genetic mutations may improve the overall quality of life for patients.

## Figures and Tables

**Table 1:** Description of the different cognitive changes and the related mutations in ALS and FTD patients.

Study Label	Gen Affected	Sociodemographic Characteristics of the Sample	Cognitive Changes
Abbate et al. [18]	*C9orf72seq* (n = 1)	A 69-year-old right-handed man with 13 years of education, presenting with subjective memory complaints. He had FTLD with prodromal hyposmia and predominant semantic deficits.	Global Cognition: Initially, global cognition was nearly normal but later declined. The patient experienced significant memory impairments, with a notable drop in MMSE scores from 29/30 to 22/30 and severe deficits in semantic and episodic memory. Language (Picture Naming Task and Achener Aphasia Test): The patient had significant language difficulties, especially with naming tasks and the comprehension of single words, while maintaining fluent speech. Memory: Both short-term (Digit Span Forward) and long-term memory were affected. Anterograde and retrograde amnesia were evident, impacting the patient’s ability to recall recent and past information (Rey–Osterrieth Complex Figure). Attention: The patient showed preserved short-term attention but experienced difficulties with tasks requiring sustained attention and complex processing (TMT, Digit Span Backward, and Stroop). There was evidence of temporal and contextual disorientation, indicating some challenges in managing attention in various contexts. Behaviour and Emotion: Behavioural changes included apathy, social withdrawal, and compulsive gambling. The patient also exhibited religious delusions and a lack of empathy.
Alcolea et al. [19]	*TDP* = 15; Control = 58	FTDL patients: *TDP*: Mean age of 61.4 years (SD = 6.5). Female sex: 53.3%. Disease duration: 3.9 years (SD = 3.4). Control: Mean age of 58.7 years (SD = 8.0). Female sex: 60.1%. Disease duration: not applicable.	Global cognition: *TDP* Participants showed lower scores in MMSE compared to healthy controls.
Arighi et al. [20]	*C9orf72seq* (n = 3)	Three case reports. Patient 1, a 53-year-old male, was diagnosed with Alzheimer’s disease. Patient 2, a 60-year-old female, was diagnosed with frontotemporal dementia. Patient 3, a 46-year-old male, was diagnosed with a condition related to frontotemporal lobar degeneration.	Patient 1 Global Cognition: Initially presented with apathy and mood changes, evolving into a deterioration of all cognitive domains, particularly prefrontal executive functions, with an MMSE score of 28/30. Language: Non-fluent aphasia, including poor speech production and simple phrases, was noted. Behavioral Assessment: Significant behavioural changes with apathy, social withdrawal, irritability, and episodes of not recognizing relatives. Visuospatial Skills: Impairment observed in visuoconstructional skills. Emotion: Loss of interest and apathy were prominent. Patient 2 Global Cognition: Moderate cognitive impairment in attention, executive functions, memory, language, and visuospatial abilities, with an MMSE score of 15/30. Language: Complete aphasia noted at the last examination. Behavioral Assessment: Severe behavioural disturbances, including agitation, aggressiveness, and suspiciousness, requiring continuous supervision. Visuospatial Skills: Significant impairment impacting both simple and complex daily activities. Emotion: Personality changes with increased suspicion and jealousy. Patient 3 Global Cognition: Mild deterioration in memory, calculation, planning, and frontal efficiency; MMSE not specified. Language: Minor issues noted but not the main concern. Behavioural Assessment: Developed mystic delusions, hallucinations, anxiety, and agitation. Visuospatial Skills: Not specifically mentioned; overall cognitive decline present. Emotion: Increased anxiety and irritability.
Barker et al. [21]	MAPT (n = 23) GRN (n = 15) C9orf72seq (n = 19) and Control (n = 143)	FTDL patients: MAPT: Mean age of 48.4 years (SD = 9.9). The group comprises 14 males and 9 females. GRN: Mean age of 61.9 years (SD = 9.9). The group comprises 7 males and 8 females. C9orf72seq: Mean age of 56.6 years (SD = 9.2). The group comprises 6 males and 13 females. Controls: Mean age of 49.5 years (SD = 11.7). The group comprises 57 males and 86 females.	*GRN*: They exhibited significantly lower scores on the MoCA, indicating greater global cognitive impairment compared to healthy controls. In terms of episodic memory, they scored lower on the Benson Figure Copy, although differences were not significant when compared to MAPT carriers. GRN carriers demonstrated a poorer performance on Trails B, showing longer times and more errors, reflecting notable impairments in executive functioning. Discriminability in recognition tasks was decreased but not significantly different from controls. There were no significant differences in intrusions on the CVLT-SF compared to non-carriers. *MAPT*: *MAPT* mutation carriers showed significantly lower MoCA total scores, indicating extensive global cognitive impairment. They had a poorer performance on the CVLT-SF, including lower scores in immediate recall, delayed recall, percent retention, and cued recall, compared to non-carriers. In recognition tasks, *MAPT* carriers had difficulties in discriminating between targets and distractors and made more false positive errors. They also performed worse on the MINT naming test compared to *C9orf72seq* carriers and non-carriers. On the Benson Figure, *MAPT* carriers recalled less information, though Craft Story results were inconclusive. *C9orf72seq*: *C9orf72seq* mutation carriers had significantly lower MoCA scores, suggesting considerable global cognitive impairment. They showed slower processing speed on Trails A, indicating potential issues in this domain. *C9orf72seq* carriers performed better on the MINT naming test compared to *MAPT* carriers, but there were no significant differences in recognition tasks. Their performance on the Benson Figure Copy was comparable to non-carriers and *MAPT* carriers, with no significant differences. Overall, their global cognitive impairment was significant, but specific impairments varied across different cognitive domains.
Beck et al. [22]	*GRN* (n = 8), *MAPT* (n = 9)	The clinical diagnoses in these patients were as follows: bvFTLD (including two with FTD-MND), SD, PNFA, 23 CBS and PSP.	MAPT: The cognitive profile was marked by significant behavioural changes such as disinhibition, apathy, and social inappropriateness, with relatively less emphasis on specific language deficits. Language and Speech: Although patients with *MAPT* mutations can experience language difficulties, these are generally less pronounced compared to those with *GRN* mutations. GRN. Episodic Memory: Patients were assessed using the Recognition Memory Tests for Words and Faces; 70% showed significant deficits, indicating genuine episodic memory impairment, often accompanied by self-reported amnestic symptoms. Language: Naming and comprehension were tested using the Graded Naming Test, Oldfield Naming Test, and Warrington Synonyms Test. Patients exhibited a range of language impairments, including dynamic aphasia, progressive mutism, and features of primary non-fluent aphasia (PNFA). Spelling and Calculation: The Graded Difficulty Spelling Test and Graded Difficulty Arithmetic Test were used to assess these skills. Deficits were observed, although specific results were not detailed. Visuospatial and Visuoperceptual Skills: These were evaluated using subtests from the Visual Object and Spatial Perception (VOSP) battery or the Block Design subtest of the WAIS-R. Significant impairments were found, particularly related to parietal lobe dysfunction.
Benussi et al. [23]	*MAPT* (n = 39), *GRN* (n = 78), *C9orf72seq* (n = 115)	Data on the patients were extracted from the GENFI dataset. All patients had symptomatic FTD. *C9orf72seq*: Mean age at symptom onset of 59.0 years (IQR = 53.0–65.0). The average years of education is 13.0 (IQR = 11.0–15.0). The group comprises 37.4% females. *GRN*: Mean age at symptom onset of 60.0 years (IQR = 55.0–66.0). The average years of education is 12.0 (IQR = 8.0–15.0). The group comprises 51.3% females. *MAPT*: Mean age at symptom onset of 52.0 years (IQR = 45.0–56.0). The average years of education is 13.0 (IQR = 11.0–16.0). The group comprises 35.9% females.	*C9orf72seq*: In the early stages of the disease, depression is the most common neuropsychiatric symptom but declines over time. Hallucinations, particularly auditory and visual, become more prominent in intermediate and late stages, surpassing other symptoms. Tactile hallucinations remain infrequent. Overall, other symptoms plateau in severity. *GRN*: Anxiety and depression are prominent in both early and late stages, with severity peaking later in the disease. Delusions and hallucinations are less frequent compared to C9orf72seq, with visual hallucinations being the most severe type but less pronounced than in C9orf72seq carriers. *MAPT*: Anxiety and depression are common in the early stages but less severe compared to other gene variants. Anxiety increases steadily over time, while depression rises mainly in the later stages. Hallucinations and delusions are notably less frequent, with visual hallucinations being the primary type but still less severe than in other gene variants.
Block et al. [24]	*GRN* (n = 1), *C9orf72seq* (n = 4), *MAPT* (n = 1)	Five case reports. A group of patients includes a 62-year-old woman with OCD diagnosed with bvFTD, a 60-year-old man with hallucinations diagnosed with FTD-MND, a 55-year-old man with delusions diagnosed with both FTLD and Alzheimer’s disease, a 53-year-old woman with mania diagnosed with ALS, and a 57-year-old man with cognitive and emotional complaints diagnosed with FTD.	Global Cognition: Global cognition appears to be more severely affected in GRN mutations compared to *C9ORF72SEQ*, where there is greater variability in the degree of impairment. Memory: Mutations in *C9ORF72SEQ* exhibit variability in memory impairment, ranging from intact to significantly deteriorated. In the case of *GRN*, although not explicitly detailed, it is likely that significant memory deterioration is present, given the overall dysfunction observed. Attention and Executive Function: Both groups show impairment, although *GRN* mutations seem to be associated with more severe and global executive dysfunction, while *C9ORF72SEQ* mutations present a broader range of deterioration, from mild to marked. Language and Communication: Mutism in *GRN* suggests a more severe impact on language and communication, whereas in *C9ORF72SEQ*, the impact is more variable and not as pronounced in most cases. Affect and Behaviour: Mutations in both genes appear to influence social and emotional cognition, although manifestations are more heterogeneous in *C9ORF72SEQ*.
Bocchetta et al. [25]	*GRN* (n = 160), *C9orf72seq* (n = 160), 67 MAPT (n = 67), non-carrier cognitively normal controls (n = 240)	*C9orf72seq* Expansion Carriers: This group consists of individuals with a mean age of 63.5 years (SD = 7.4). Non-Carriers: Have a mean age of 44.8 years (SD = 12.2), with 42.9% being male. *MAPT* Mutation Carriers: This group has a mean age of 41.1 years (SD = 10.6). Gender distribution details are not provided. *GRN* Mutation Carriers: Have a mean age of 59.2 years (SD = 9.3), and 40.4% are male. The clinical phenotypes include 36 cases of bvFTD, 4 FTD-ALS, 2 ALS, 2 PPA, 1 PSP, 1 Dementia-NOS, and 1 Other.	Across all genetic groups, presymptomatic carriers with normal baseline brain region W-scores did not show a substantial progression in clinical, cognitive, or behavioural scores after 12 months. Significant changes were observed but were minimal: less than 1 point on the CDR^®^ plus NACC FTLD total score and less than 2 points on the CBI-R total score, with values lower than those in abnormal groups. The only exception was the MD for the sCC in *C9orf72seq* expansion carriers, which showed a 3-point increase on the CDR^®^ plus NACC FTLD total score, comparable to abnormal groups. Diffusion measures indicated slightly larger significant differences in progression compared to GM volumes, especially for *MAPT* and *C9orf72seq* mutation carriers regarding behavioural scores. For GRN mutation carriers, progression was similar between GM and WM regions. Specific regional measures showed larger increases in clinical scores. *C9orf72seq* and *GRN* mutation carriers experienced a similar worsening in behavioural symptoms, up to 8–10 points. *MAPT* mutation carriers showed a worsening up to 11 points, potentially up to 19 points in some regions if confirmed in larger samples. However, GRN and *C9orf72seq* carriers had a greater increase in the CDR^®^ plus NACC-FTLD sum of boxes scores (4–5 points) compared to *MAPT* carriers (2 points), which were not statistically significant. This difference may relate to the CDR^®^ plus NACC-FTLD capturing more cognitive and linguistic features relevant to *GRN* and *C9orf72seq* mutation carriers compared to the CBI-R.
Borroni et al. [26]	*TARDBP* (n = 1)	A 74-year-old female who develop bvFTD without MND carrying a pathogenetic mutation within *TARDBP*, namely N267S	Global Cognition: At baseline, the patient had an MMSE score of 20, which declined to 16 over three years. The decline in overall cognitive function is consistent with the progressive nature of the disease. Memory: Neuropsychological tests showed a stable performance in story recall with minor fluctuations. Immediate recall of complex figures (Rey Figure) was very poor, indicating significant memory impairments. Language: Language comprehension declined progressively over three years. Despite initial stability in language function, there was a noticeable deterioration in tests assessing language skills. Attention and Executive Function: Executive function was significantly impaired, with difficulties observed in tasks like the Trail Making Test and verbal fluency. There was a consistent decline in cognitive flexibility and problem-solving abilities. Visuospatial Skills: Visuospatial abilities were notably impaired, as evidenced by poor performance on the Rey Figure copy and recall tasks. However, basic visual perception remained intact. Emotional and Behavioural Disorders: The patient exhibited apathetic behaviour and persistent behavioural disturbances, with no significant changes reported. Emotional distress remained relatively stable, without signs of depression. Functional Abilities: Instrumental Activities of Daily Living (IADLs) worsened progressively, leading to complete dependence. Basic Activities of Daily Living (BADLs) also showed a gradual decline.
Boutoleau-Bretonnière et al. [27]	*SQSTM1* (n = 3)	Three case reports were identified from a French family: the proband and two siblings, 77, 71, 74 years old, diagnosed with bvFTD.	Patient 007: The patient showed speech apraxia, dysarthria, and mild grammatical issues, but comprehension was intact. He had significant executive dysfunction and visuoconstructional impairments, with preserved memory. Over time, his phonetic and grammatical difficulties worsened, but comprehension remained stable. Patient 008: This patient had speech apraxia and moderate dysarthria, with preserved autonomy. He experienced significant executive dysfunction and visuoconstructional deficits but no aphasia. Imaging showed major frontal and parietal atrophy and severe hypoperfusion. His condition slowly progressed, affecting executive functions but allowing continued daily activity. Patient 009: The patient experienced hallucinations, preserved memory, but severe executive dysfunction and visuoconstructional difficulties. Behavioural problems, including self-mutilation, developed over time. His condition progressed to bvFTD, leading to institutionalisation and eventual death.
Bouzigues et al. [28]	*C9orf72seq* (n = 54) *GRN* (n = 26) *MAPT* (n = 21) and controls (n = 248)	FTDL’s patients: *MAPT*: Mean age of 57.3 years (SD = 10.2). The group comprises 33.3% females. *GRN*: Mean age of 63.5 years (SD = 7.9). The group comprises 48.8% females. *C9orf72seq*: Mean age of 62.1 years (SD = 8.6). The group comprises 34.8% females. Controls: Mean age of 44.9 years (SD = 12.7). The group comprises 56.8% females.	Global Cognition: The *GRN*, *MAPT* and *C9orf72seq* groups had a significantly lower MMSE total score, suggesting more global cognitive impairment. Language: All three mutation groups exhibited notable challenges in naming tasks, with MAPT carriers showing the most pronounced deficits.
Bradfield et al. [29]	*FUS* (n = 1)	A 61-year-old patient with frontotemporal dementia (FTD) associated with frontotemporal lobar degeneration (FTLD)	Global Cognition: The patient’s global cognition declined progressively, with MMSE scores dropping from 27/30 in late 2013 to 25/30 in early 2015 and AMTS scores from 8/10 to 6/10, reflecting worsening cognitive function. Language: Language abilities deteriorated, showing significant impairments in word recall and phonemic fluency, consistent with semantic dementia. Anomia and semantic errors were noted, but prosopagnosia was absent. Memory: Memory issues included severe problems with word recall and episodic memory, though short-term memory remained relatively intact. Attention and Executive Functioning: The patient had difficulties with executive functions and planning, supported by neuroimaging findings of frontal and temporal lobe hypometabolism and cortical atrophy. Behaviour and Emotion: Behavioural changes included increased disinhibition, impulsivity, and obsessive behaviours, with notable social withdrawal and personality changes, indicative of bvFTD.
Bussy et al. [30]	*MAPT* (n = 24), *GRN* (n = 44), *C9orf72seq* (n = 62), Controls (n = 281)	FTDL patients from the GENFI2 dataset. *MAPT*: Mean age of 58.5 years (SD = 8.23). The group comprises 33.3% females. *GRN*: Mean age of 63.2 years (SD = 7.07). The group comprises 56.8% females. *C9orf72seq*: Mean age of 63.7 (SD = 7.23). The group comprises 40.3% females. Controls: Mean age of 46.1 years (SD = 13.5). The group comprises 58.4% females.	Behavioural Assessment (CBI-R): Symptomatic individuals exhibited higher scores across various behavioural domains compared to controls, indicating more frequent and severe behavioural deficits. The CBI-R assessed areas like memory, everyday skills, mood, and abnormal behaviour, with scores of 3 or 4 signifying severe impairments, which were more prevalent in symptomatic individuals. Presymptomatic individuals exhibited behaviour and cognitive function similar to controls, with no marked deficits detected in the early stages.
Byrne et al. [31]	*C9orf72seq*+ (n = 21) *C9orf72seq*- (n = 170)	A population-based register of patients with ALS in Ireland. Patients without the C9orf72seq repeat expansion (n = 170): Mean age at onset of 61.3 years (SD = 10.6) and mean age at diagnosis of 62.5 years (SD = 10.6). The group comprises 40.6% females. Patients with the C9orf72seq repeat expansion (n = 21): Mean age at onset of 56.3 years (SD = 8.3) and mean age at diagnosis of 57.5 years (SD = 8.1). The group comprises 52.4% females.	Language. There was no significant difference in verbal fluency between patients with and without the C9orf72seq repeat expansion. Patients with the C9orf72seq repeat expansion tended to have higher scores, indicating somewhat better verbal fluency, though the difference was not statistically significant. Cognitive Flexibility (Brixton Test): Patients without the C9orf72seq repeat expansion showed significantly better cognitive flexibility compared to those with the expansion. Executive Control (Stroop): Patients without the C9orf72seq repeat expansion had higher scores, suggesting more difficulty with executive control, though the result was near significance. Working Memory (Backward Digit Span): No significant difference was found.
Caso et al. [32]	*C9orf72seq* (n = 2)	Two case reports: A 60-year-old woman and a 64-year-old man who had a temporal variant of frontotemporal dementia.	Case Report 1: Stable global cognition initially (MMSE 29, CDR 0.5) but significant impairments in executive functions, visuospatial abilities, and memory over a year. Severe deficits in language, particularly in picture naming and reading. Social cognition was notably impaired, with deficits in empathy and theory of mind. Case Report 2: Moderate initial cognitive impairment (MMSE 26, CDR 2), with some improvement in the CDR score to 1. Severe declines in executive functions, visuospatial abilities, and memory. Extremely poor performance in visual semantic memory and language tasks. Significant social cognition deficits, including empathy and theory of mind, with high psychiatric symptom levels.
Castelnovo et al. [33]	*GRN* c.1018delC (p.H340TfsX21) mutation (n = 1)	A 60 years-old white right-handed patient with 8 years of education, who at onset complained about prevalent language production difficulties, such as effortful speech and anomia.	The patient demonstrated a notable cognitive decline over time, as observed from 24 to 31 months post-symptom onset: Global Cognition: MMSE scores dropped from 21 to 19, indicating worsening global cognition, remaining below the cut-off of 23.8. FAB scores were consistently low, indicating significant frontal lobe dysfunction. Language Abilities: Confrontation naming and Object knowledge deteriorated, showing deficits by 31 months. Repetition, Reading, and Writing abilities were impaired by 24 months and further declined by 31 months. Syntactic comprehension showed deficits at both time points. Memory: Digit Span Forward remained stable but at a borderline level, while spatial span forward worsened. Benson’s figure recall dropped to zero by 31 months, indicating severe memory impairment. Executive Functions: Attentive matrices scores fell significantly, showing a marked decline in attention and executive functioning. Digit Span Backward showed a borderline performance, with no further testing at 31 months. Social Cognition: The patient had deficits in global social cognition and intention attribution at both time points. Emotion attribution improved slightly but remained borderline. Visuospatial Abilities and Praxia: Benson’s figure copy remained stable, while copy of simple figures showed slight impairment by 31 months. Orofacial apraxia and ideomotor apraxia scores indicated significant motor planning deficits at both time points. Mood, Autonomy, and Disease Severity: Neuropsychiatric inventory scores were low, with no significant neuropsychiatric symptoms reported. Frontal behavioural inventory and CDR scores indicated progressive frontal and dementia-related behaviours.
Chiò et al. [34]	*C9orf72seq* (n = 37), *TARDBP* (n = 12), *SOD1* (n = 6), FUS (n = 2) *OPTN* (n = 2), *MATR3* (n = 1)	Patients diagnosed with ALS.	*C9orf72seq* expansion was the strongest determinant of both comorbid FTD and, to a lesser degree, one of the intermediate forms of cognitive impairment (ALS-Ci) but not ALS-Bi. Global cognition: Patients with *C9ORF72SEQ* mutations exhibited a range of cognitive impairments. The cognitive testing revealed a significant proportion of these patients had normal cognition, but a notable subset displayed various levels of cognitive decline.
Christidi et al. [35]	*C9orf72seq* (n = 22); Control (n = 111)	All patients had ALS. C9-ALS (n = 182): Mean age is 61.57 years (SD = 12.28). The group comprises 65.9% males. C9+ALS (n = 22): Mean age is 58.00 years (SD = 8.98). The group comprises 63.6% males. HC (n = 111): Mean age is 59.55 years (SD = 10.81). The group comprises 48.6% males.	Global Cognition: Based on the ECAS total score, global cognitive function appears similar between C9-ALS and C9+ALS patients. Memory: The ECAS—Memory score indicates that memory impairment is more pronounced in C9+ALS patients compared to C9-ALS. Executive Function: While C9+ALS patients show a trend towards greater executive dysfunction, the difference between the groups is not definitive. Language and Verbal Fluency: Both groups demonstrate similar levels of performance in language and verbal fluency as measured by the ECAS—Language and ECAS—Verbal Fluency scores. Visuospatial Functions: Visuospatial abilities, as assessed by the ECAS—Visuospatial Functions score, are comparably maintained in both groups.
Chu et al. [36]	*MAPT* (n = 6), Control (n = 30)	Presymptomatic behavioural variant frontotemporal dementia (bvFTD) patient. *MAPT*. Mean age of 49 years (SD = 3.90). The group comprises 50% females. Control. Mean age of 56.57 years (SD = 9.64). The group comprises 53.3% females.	Global Cognition: Presymptomatic *MAPT* patients had similar scores to the control group on the MMSE, MoCA, and FTD-CDR sum of boxes. Language: Presymptomatic *MAPT* patients had similar scores to the control group on the BNT. Behavioural Assessment: Presymptomatic *MAPT* patients scored higher than healthy controls on the NPI and FBI.
Clarke et al. [37]	*MAPT* (n = 6), Control (n = 12)	Presymptomatic FTD patients from the GENFI dataset. *MAPT*. Mean age of 44.8 years (SD = 6.3). The group comprises 16.7% females. Control. Mean age of 46.1 years (SD = 7.2). The group comprises 50% females.	Global Cognition: Presymptomatic *MAPT* patients had similar scores to the control group on the MMSE, MoCA, Frontal Assessment Battery, and FTD-CDR sum of boxes.
Colombo et al. [38]	*C9orf72seq* (n = 55)	A cohort of 960 Italian patients diagnosed with amyotrophic lateral sclerosis (ALS) and other motor neuron diseases, according to the El Escorial revised criteria, was consecutively recruited at the IRCCS Istituto Auxologico Italiano, a tertiary ALS centre in Milan, Italy, between 2008 and 2021. Of these patients, 37.2% (357) were female and 62.8% (603) were male, with a mean age at onset of 59.3 years (±12.3). *C9orf72seq* hexanucleotide repeat expansion (HRE) was identified in 55 (5.7%) patients, of whom 19 (18.8%) had familial ALS (FALS) and 36 (4.2%) had sporadic ALS (SALS). In terms of clinical presentation, 495 patients (51.8%) exhibited classic ALS, 204 (21.4%) had bulbar ALS, and 19 (2.0%) had respiratory ALS. Lower motor neuron (LMN)-predominant phenotypes included 42 (4.4%) patients with progressive muscular atrophy (PMA), 42 (4.4%) with flail arm syndrome, and 24 (2.5%) with flail leg syndrome. Additionally, 89 (9.3%) patients were diagnosed with upper motor neuron (UMN)-predominant ALS and 40 (4.2%) with primary lateral sclerosis (PLS).	The comparison of cognitive and behavioural features between *C9orf72seq*-positive (C9Pos) and *C9orf72seq*-negative (C9Neg) patients revealed several key findings: Cognitive Function: No significant differences were found between C9Pos and C9Neg patients across various cognitive domains, including executive functions, fluency, language, ALS-specific and ALS-nonspecific cognitive scores, memory, visuospatial abilities, and the overall ECAS total score. Behavioural Symptoms: C9Pos patients exhibited a significantly higher prevalence of disinhibition compared to C9Neg patients. Additionally, C9Pos patients had significantly higher scores on the FBI-B subscale (which measures behaviours such as disinhibition, apathy, and loss of empathy), indicating greater behavioural impairment in this group. However, no significant differences were observed between the two groups for apathy/inertia, loss of sympathy/empathy, perseverative/stereotyped behaviour, hyperorality, or overall ECAS behavioural symptoms. Depressive Symptoms: C9Neg patients scored significantly higher on the Beck Depression Inventory-II (BDI-II), both in cognitive–affective and somatic subscales, as well as on the total BDI-II score, indicating a greater severity of depressive symptoms in this group. Anxiety Levels: C9Neg patients also reported significantly higher levels of anxiety, as measured by the State–Trait Anxiety Inventory (STAI), compared to C9Pos patients.
Devenney et al. [39]	C9orf72seq (n = 10); Controls (n = 35)	FTD’s patients. *C9orf72seq*: Mean age of 54.1 years (SD = 9.4). The group comprises 40% females. Controls: The group comprises 51.4% females.	Global Cognition: Carriers and non-carriers showed cognitive and behavioural deficits compared to controls, with non-carriers exhibiting more severe impairment. Visuospatial Skills: *C9ORF72SEQ* mutation carriers scored significantly lower on the copy subscale of the Rey–Osterrieth Complex Figure Test compared to non-carriers, indicating difficulties in accurately reproducing complex visual information. No significant differences were observed between mutation carriers and non-carriers in VOSP, suggesting that basic visual object and spatial perception skills were similar across both groups.
Devenney et al. [40]	C9orf72seq (n = 14), Controls (n = 23)	bvFTD or FTD-ALS patients. *C9orf72seq*: Mean age of 61.2 years (SD = 5.9). The group comprises 21.4% females. Controls: Mean age of 62.5 years (SD = 3.9). The group comprises 39.1% females.	Global cognition: Controls had higher scores on both the ACE-R and FRS compared to C9orf72seq patients, indicating better cognitive performance. Perceptual Disorders (NPI): The psychosis index was higher in C9orf72seq carriers (64% showed psychotic symptoms) compared to non-carriers (26%).
Devenney et al. [41]	C9orf72seq (n = 16), Control (n = 16)	Patients with the *C9orf72seq* mutation had ALS (n = 28), ALS-Plus (n = 9), ALS-FTD (n = 11), or bvFTD (n = 27), with 76% of them being women. Their average age ranged between 60 and 63 years. Controls: Mean age of 60 years (SD = 10.5). The group comprises 48% females.	Global cognition: Controls had higher scores on both the ACE-III and FRS compared to *C9orf72seq* patients. Perceptual Disorders: *C9orf72seq* carriers experienced more frequent and severe perceptual disorders compared to non-carriers and controls.
Dong et al. [42]	*MAPT* (n = 7) *TBK1* (n = 7) *GRN* (n = 2) *GRN*+*TBK1* (n = 1) *VCP* (n = 1) *TARDBP* (n = 1) *UBQLN2* (n = 1) *SQSTM1* (n = 1) *DCTN1* (n = 1) *HNRNPA1* (n = 10) *C9orf72seq* (n = 1)	204 unrelated participants of Chinese ancestry were enrolled from the PUMCH dementia cohort between 2007 and 2021. 52.0% (106/204) were males and 48.0% (98/204) were females. 11.8% (24/204) of participants harboured the potential causative variants in FTD-related genes. Of them, 3.5% subjects had the *MAPT* variants, and 3.5% had the TBK1 variants. The remaining 10 cases carried the rare variants in *GRN*, *GRN*+*TBK1*, *VCP*, *TARDBP*, *UBQLN2*, *SQSTM1*, *DCTN1*, and *HNRNPA1*, as well as GGGGCC repeats in the *C9orf72seq*.	*C9orf72*. Carriers of the GGGGCC repeat expansion in the C9orf72 gene predominantly presented with early behavioural and psychiatric symptoms, leading to a diagnosis of behavioural variant frontotemporal dementia (bvFTD). This profile was associated with a high incidence of a family history of dementia. Over the course of the disease, some individuals developed motor symptoms or language deficits. *TBK1*. Carriers of mutations in *TBK1* exhibited varied symptoms, with some cases starting with stereotyped behaviours or loss of empathy, and others presenting with aphasia or language difficulties. Over time, several developed motor neurone disease (MND) or motor dysfunction. *MAPT* Mutations in *MAPT* were associated with an earlier onset of disease, typically featuring severe behavioural and language symptoms. All carriers displayed frontal atrophy on neuroimaging. These cases were more often hereditary and frequently showed marked behavioural symptoms, such as disinhibition or compulsive behaviours, alongside progressive aphasia. *TARDBP*. Carriers of mutations in *TARDBP* were characterised by early-onset semantic aphasia and compulsive behaviours, such as excessive smoking or picking up cigarette butts. These individuals often had a family history of language impairment and psychotic disorders. *GRN*. Mutations in *GRN* were associated with early non-fluent or semantic aphasia. Some individuals also developed behavioural symptoms such as disinhibition, loss of empathy, and compulsive behaviour. In certain cases, mutations in both *GRN* and *TBK1* were found, suggesting a more complex phenotypic presentation. Neuroimaging showed predominant atrophy in the left temporal lobe. *UBQLN2*. Carriers of mutations in *UBQLN2* began with symptoms such as apathy, loss of empathy, and dietary changes, followed by rapid progression to MND. These cases had a family history of schizophrenia and psychiatric disorders, indicating a possible link with severe neuropsychiatric symptoms. *SQSTM1*. Carriers of mutations in *SQSTM1* presented with early semantic aphasia, exhibiting a cognitive profile similar to other mutations affecting language. *VCP*, *DCTN1*, *HNRNPA1*. Mutations in less common genes, such as *VCP*, *DCTN1*, and *HNRNPA1*, were primarily associated with early-onset semantic or non-fluent aphasia, followed by repetitive behaviours, parkinsonism, or behavioural changes.
Downey et al. [43]	C9orf72seq (n = 5), MAPT (n = 7), Controls (n = 13)	FTD patients. *C9orf72seq*: Mean age of 65 years (SD = 8). *MAPT*: Mean age of 62 years (SD = 4). Both groups are comprised of 0% females. Controls: Mean age of 62 years (SD = 5). The group comprises 23.1% females.	*C9orf72seq* Mutation Carriers: C9orf72seq mutation carriers exhibited significant impairments in global cognition on the MMSE compared to healthy controls. In terms of language, they performed better on naming tasks (GNT) compared to MAPT-FTD and sporadic-FTD groups, with no differences observed in BPVS. All FTD groups, including *C9orf72seq*-FTD, showed impairments in episodic (RMT) and semantic memory (GNT), but short-term memory remained relatively intact. Social cognition was also impaired, with no significant differences among the FTD groups. Visuospatial skills were generally preserved and similar to healthy controls. *MAPT* Mutation Carriers: MAPT mutation carriers had significant global cognitive impairments on the MMSE compared to healthy controls. They performed worse on naming tasks (GNT) than *C9orf72seq*-FTD, although BPVS scores did not differ. All FTD groups, including MAPT-FTD, showed impairments in episodic (RMT) and semantic memory (GNT), with short-term memory remaining relatively unaffected. Social cognition was compromised across all FTD groups, with no significant differences between the groups. Visuospatial skills were largely spared and comparable to those of healthy controls.
Finger et al. [44]	*C9orf72seq* (n = 17) *MAPT* (n = 9) *GRN* (n = 16)	Ninety-two young adults in the GENFI study met the inclusion criteria and were designated as presymptomatic (unaffected) by their local site physicians. The Frontotemporal Lobar Degeneration Clinical Dementia Rating (FTLD-CDR) global rating was 0 for all but five participants, who received ratings of 0.5; among these, two were mutation carriers and three were non-carriers. The mean age at the time of participation was 25 years (range: 19–29), and the mean level of education was 14 years (range: 8–18). All of these young adults were classified as unaffected/presymptomatic by the site physicians.	No significant differences between *C9orf72seq* repeat expansion carriers versus non-carriers were observed in the other behavioural scales or cognitive tasks. *MAPT* mutation carriers performed better than non-carriers on verbal fluency (letter) performance and Digit Span Forward. *GRN* mutation carriers performed better on the digit symbol task than non-carriers.
Floeter et al. [45]	C9orf72seq (n = 21), Controls (n = 28)	Patients with the *C9orf72seq* mutation had ALS (n = 11), ALS-FTD (n = 7), or bvFTD (n = 3), with 76% of them being women. Their average age ranged between 52.4 and 61.9 years. The group comprised 28.6% females. Controls: Mean age of 52.8 years (SD = 9.1). The group comprises 35.8% females.	Global Cognition: The MMSE scores were lower in *C9*+ ALS-FTD and bvFTD patients compared to other groups, indicating significant cognitive impairment in these subgroups. Language: Letter fluency scores were lower in *C9*+ ALS-FTD and bvFTD patients, suggesting executive function deficits. Behavioural Assessment: The FBI scores were lower in *C9*+ ALS-FTD and bvFTD patients, which is consistent with the cognitive-behavioural impairments typical of these conditions.
Floris et al. [46]	*C9orf72seq* (n = 8)	Our cohort included 32 males and 24 females; 30 patients presented with behavioural variant FTD (bvFTD), six with progressive non-fluent aphasia (PNFA), seven with semantic dementia (SD), six with unspecified primary progressive aphasia (PPA), and seven with a mixed form of FTD (bvFTD PPA). A familial positive history for FTD and/or ALS was recorded in 19 patients (34%) whereas 37 cases were sporadic. The *C9orf72seq* mutation was detected in 8 out of 56 (14.2%) index cases and in 6 out of 19 (31.6%) familial cases. Clinically, seven patients had bvFTD and two a mixed form of FTD. *C9orf72seq* patients had a younger age of onset (median age 58 vs. 67 years, *p* 0.01) and a higher frequency of a positive familial history for FTD/ALS (7/9 (77.8%) vs. 7/27 (25.9%).	Neuropsychological tests assessing executive functions, working memory, and verbal and visuospatial long-term memory domain failed to discriminate between *C9orf72seq*+ and *C9orf72seq*—patients. The *C9orf72seq* mutation carriers scored significantly worse than the non-carriers on tests assessing constructional apraxia and on word–picture matching tests for category nouns. *C9orf72seq* differed from *C9orf72seq* patients in the higher frequency of delusional psychotic symptoms and hallucination. No differences were found in apathy, disinhibition, aggressivity, agitation, euphoria, irritability, repetitive behaviour, and eating disorders.
Foster et al. [47]	*C9orf72seq* (n = 65), 193 *GRN* (n = 47), *MAPT* (n = 57), Controls (n = 216)	Data on the patients were extracted from the FTD Initiative (GENFI) dataset. All patients had symptomatic FTD. *MAPT*: Mean age of 58.9 years (SD = 9.4). The group comprised 57% males. *GRN*: Mean age of 63 years (SD = 7.4). The group comprised 47% males. *C9orf72seq*: Mean age of 62.9 (SD = 9.5). The group comprised 37.5% females. Controls: Mean age of 45.7 (SD = 13). The group comprised 40% males.	Global Cognition: All FTD patient groups showed significantly lower scores in MMSE and CDR plus NACC FTLD-SB compared to healthy controls. Social Cognition (mIRI): All FTD patients in all genetic groups scored worse on all three measures of empathy than controls.
Foxe et al. [48]	*C9orf72seq* (n = 2)	Two case reports. LS, an individual in their late 40s with 12 years of formal education, was diagnosed with a mixed presentation of FTD and MND. AS, second-degree relative of LS with 15 years of formal education, presented in their early 80 s, 5 years after the onset of cognitive and behavioural symptoms.	Case 1 (LS) Global Functioning: LS’s general cognitive abilities, as assessed by the ACE–III, declined significantly from 85 to 57 over three years, indicating worsening overall impairment. Executive Function: Severe declines were observed in executive functions, with substantial increases in completion times for the Trail Making Test and Hayling Sentence Completion Test. Learning and Memory: Memory deteriorated markedly, with severe reductions in recall and learning abilities as shown by the RCFT and RAVLT. Language: Language skills dropped sharply, especially in naming and repetition, as evidenced by reduced scores on the Sydney Language Battery. Visuospatial Skills: Visuospatial abilities remained relatively stable with minor changes in Clock Drawing performance. Emotion Processing and Behaviour: LS experienced mood stability but exhibited significant behavioural changes, including decreased self-care and sleep issues, as reflected in the CBI–R and NPI. Case 2 (AS) Global Functioning: AS’s cognitive abilities declined moderately, with ACE–III scores falling from 70 to 64 over a year, showing a gradual decrease in overall function. Executive Function: AS showed significant impairments in executive functions, with reduced scores on tasks like Animal Fluency and the Trail Making Test. Learning and Memory: AS had severe difficulties with learning and memory, demonstrated by poor performance on the Digit Span and RAVLT. Language: Language abilities were notably impaired, particularly in naming and fluency, with difficulties in speech and written sentences. Visuospatial Skills: Visuospatial skills declined slightly, with reduced performance on Clock Drawing. Emotion Processing and Behaviour: AS showed reduced emotion processing and behavioural rigidity, with a moderate level of depression and stress, impacting daily activities and functional capacity.
Gabryelewicz et al. [49]	*PGRN* (n = 2)	The patient 1 was a 65-year-old male diagnosed with Frontotemporal Dementia with Parkinsonism (FTDP). His condition was marked by significant cognitive and behavioral decline. His brother (patient), also male, was initially diagnosed with Frontotemporal Dementia with Progressive Non-fluent Aphasia (PNFA) at age 57. His diagnosis evolved over time to include behavioural variant frontotemporal dementia (bvFTD) and Corticobasal Syndrome (CBS) by age 60.	Patient 1 Global Cognition: Exhibited slowness, apathy, and significant cognitive decline with an MMSE score of 20 at age 64. Rapid deterioration, with insomnia and psychotic symptoms noted. Language: Developed aphasia with pronounced word-finding difficulties and impaired verbal learning. Behavioural Assessment: Significant personality changes including disinhibition, loss of initiative, and psychotic features such as hallucinations and bizarre delusions. Visuospatial Skills: Impaired visuospatial abilities with disorganised Rey–Osterrieth Complex Figure reproduction and difficulties in spatial praxis. Emotion: Loss of interest and initiative, irritability when opposed. Patient 2 Global Cognition: Initially presented with moderate impairments in multiple cognitive domains, evolving to severe deterioration, with an MMSE score of 9/30 and significant functional decline. Language: Initially had word-finding difficulties and non-fluent aphasia, later progressed to Broca’s aphasia. Behavioural Assessment: Developed disinhibition, impulsivity, and compulsive behaviours. Progressive behavioural and motor symptoms led to a diagnosis of bvFTD and later CBS. Visuospatial Skills: Relative sparing of visuospatial tasks early on but later developed significant deficits. Emotion: Experienced euphoria, apathy, and appetite dysregulation.
García-Roldán et al. [50]	*FUS* (n = 1)	A 37-year-old man, without a family history of neurodegenerative diseases, was brought by his family to consult for dysarthria and behavioural change. No mutations were found in genes such as *Tau*, *progranulin*, *C9orf72seq*, *FUS*, *TDP*-43, *CHMP2B*, or *VCP*. In necropsy, found positive for fused in sarcoma (*FUS*).	The changes in the behaviour of the patient were getting worse with a loss of decorum, empathy, and compulsive actions. Dysarthria worsened, and then dysphagia emerged. At 38 years old, marked utilisation behaviour and lack of insight were already evident.
Gendron et al. [4]	Controls (n = 144) Presymptomatic *C9orf72seq* or a *GRN* or *MAPT* (n = 85)	Mutation negative individuals in kindred with an FTD-causing mutation (controls; n = 144), presymptomatic individuals with a *C9orf72seq* repeat expansion or a *GRN* or *MAPT* mutation (n = 85), and patients with sporadic or genetic bvFTD (n = 289), nfvPPA (n = 72), svPPA (n = 84), CBS (n = 89), or PSP-RS (n = 124). Patients with FTD-ALS (n = 25), ALS (n = 12), and mild cognitive or behavioural changes (mild cognitive impairment [MCI]) (n = 57) were included for comparison in some analyses.	Controls: This group, which serves as a baseline, showed no cognitive impairment, with all individuals scoring a CDR+NACC-FTLD global score of 0. Presymptomatic Mutation Carriers: Similar to the controls, these individuals did not exhibit any cognitive impairment, with a CDR+NACC-FTLD global score of 0. Behavioural Variant Frontotemporal Dementia (bvFTD): The majority (51.2%) of individuals in this group scored 2 on the CDR+NACC-FTLD global scale, indicating moderate impairment. A substantial number (33.2%) scored 1, showing mild impairment. No individuals scored 0. Non-Fluent Variant Primary Progressive Aphasia (nfvPPA): The distribution here shows a significant percentage of moderate impairment (37.5% scored 2) and mild impairment (43.1% scored 1). A small number (2.8%) scored 3, indicating severe impairment. Semantic Variant Primary Progressive Aphasia (svPPA): Similar to the nfvPPA group, the svPPA group had a high proportion with moderate impairment (28.6% scored 2) and mild impairment (57.1% scored 1). A minor fraction (1.2%) showed severe impairment. Corticobasal Syndrome (CBS): This group exhibited a diverse range of cognitive impairment, with a notable proportion scoring 2 (24.7%) and 1 (42.7%), indicating moderate to mild impairment, respectively. Only a small number (3.4%) scored 3. Progressive Supranuclear Palsy–Richardson Syndrome (PSP-RS): Individuals in this category also displayed a broad range of impairment, with moderate (33.9%) and mild (39.5%) scores being prevalent. Severe impairment was noted in 8.1% of the individuals. Mild Cognitive Impairment (MCI): The MCI group had a distribution indicating mild to moderate impairment, with the majority scoring 2 (40.0%) and 1 (36.0%). Frontotemporal Dementia with Amyotrophic Lateral Sclerosis (FTD-ALS): This group had the highest proportion of severe impairment, with 25.0% scoring 3 and 41.7% scoring 1. Amyotrophic Lateral Sclerosis (ALS): The ALS group exhibited severe cognitive impairment, with 25.0% scoring 3 and 8.3% scoring 1.
Gossye et al. [3]	*MAPT* (p.R406W) (n = 28) *GRN* (n = 37) *VCP* (n = 6) *TBK1* (n = 6) *PSEN1* (n = 2) *FUS* (n = 2) *TARDBP* (n = 1) *C9orf72seq* (n = 2)	Among the FTD patients, a pathogenic *C9orf72seq* repeat expansion mutation was identified in 50 patients. Two patients carried *MAPT* mutations different from p.R406W (p.G273R and p.S305S). The p.R406W mutation was present in 13 potentially affected patients and in 12 presymptomatic carriers. Two FTD patients carried the *C9orf72seq* repeat expansion mutation and a pathogenic mutation in VCP or GRN. In the Alzheimer’s disease patients, pathogenic mutations were identified in *PSEN1* (n = 6) and APP (n = 1). Median age at death was significantly lower in patients with bvFTD (68.0 years) than in those with Alzheimer’s disease (78.5 years, *p* = 0.015).	Memory problems were reported the most, not only in those with clinical Alzheimer’s disease (100.0%) but also in those with bvFTD (88.9%). Neuropsychiatric symptoms were also common across the cohort, with most notably a high incidence of disinhibition/aggressiveness in both bvFTD (100.0%) and Alzheimer’s disease (58.3%) patients. Other neuropsychiatric symptoms and cognitive symptoms were depressiveness (42.1%), compulsive behaviour/perseveration (41.2%), symptoms compatible with executive dysfunction (47.1%), and spatial disorientation (47.4%).
Gowell et al. [51]	*FUS* (n = 1)	A 44-year-old man with FTD	Cognitive Function: The patient displayed severe global cognitive impairment with a low ACE-III score (55/100), highlighting significant deficits in various cognitive areas. Executive Function: Executive functions were critically impaired, shown by a poor performance in Letter Fluency (first centile), Trail Making Test (part A at third centile, part B discontinued), and Stroop Test (below the first centile). Verbal working memory was notably deficient (Digit Span test: 4 forward, 3 backward). Memory: Verbal memory was severely impaired, with scores at the first and second centiles for immediate and delayed recall. However, visuospatial memory was relatively preserved, with high scores in immediate and delayed recall of a complex figure (92nd and 81st centiles). Language: Language abilities showed difficulties in naming, as indicated by a Graded Naming Test score of 20/30, though sentence comprehension and grammatical production remained intact. Visuospatial Abilities: Visuospatial skills were relatively intact, with accurate performance in copying complex figures and good scores in visuospatial episodic memory. Social Behaviour and Psychopathological Symptoms: Behavioural changes included repetitive actions, disinhibited behaviour, and a new preference for sweet foods, with an initially low mood and decreased social engagement.
Gramaglia et al. [52]	*C9orf72seq* (n = 1)	A 57-year-old Caucasian woman who had bvFTD.	Global Cognition: The patient demonstrated significant cognitive decline, as shown by a lower-than-expected score on the Milan Overall Dementia Assessment (MODA). Language: Severe language impairments were noted, with a low Verbal IQ on the WAIS-R and poor performance on tests of phonemics, semantics, and reasoning. Executive Functions: Executive functions were impaired, with poor results on the FAB, particularly in Phonemic Fluency and Affinity. Attention: Attention and concentration were inconsistent, with difficulties highlighted during assessments. Behaviour: The patient exhibited bizarre behaviours, mystic delusions, and emotional instability, as confirmed by her husband’s reports and the MMPI-2 results. Emotion: Emotional dysregulation was evident, with a tendency to express psychological distress through somatic complaints.
Heuer et al. [53]	*MAPT* (n = 28), *GRN* (n = 14), *C9orf72seq* (n = 43)	All patients had familial bvFTD. *C9orf72seq*: Mean age at visit of 60.6 years (SD = 9.6). The group comprised 46.5% females. *GRN*: Mean age at visit of 65.4 years (SD = 8.2). The group comprised 57.1% females. *MAPT*: Mean age at visit of 53.8 years (SD = 8.7). The group comprised 46.4% females.	*C9orf72seq* expansion carriers showed a range of cognitive impairments, but these were generally comparable to those observed in sporadic bvFTD cases. Performance on neuropsychological tests, including executive function and working memory, did not significantly differ from the sporadic group. *GRN* mutation carriers exhibited the poorest performance on the “Number Span Forward” test compared to other groups, indicating more pronounced deficits in executive function and working memory. This group had notable impairments in span length and number of correct trials. *MAPT* mutation carriers showed cognitive impairments that were intermediate between those seen in *GRN* mutation carriers and sporadic cases. They did not have the extreme deficits observed in *GRN* carriers but performed worse than sporadic bvFTD patients.
Hokelekli et al. [54]	*C9orf72seq* (n = 1)	68-year-old right-handed male with 20 years of education, presenting with progressive cognitive and motor deficits over 15 years. He had bvFTD.	Global Cognition: Initial cognitive assessments showed normal global cognition (Short Test of Mental Status: 36/38, Dementia Rating Scale 2: 143/144). However, the patient developed more pronounced memory and executive dysfunction over time. Language: The patient had word-finding difficulties and slow, effortful speech. He scored 23/24 on a language exam, missing one point for difficulty with multi-step verbal commands. Executive Functions: The patient exhibited significant executive dysfunction, including difficulty performing sequences in his morning routine, managing finances, and multitasking. He developed compensatory mechanisms to manage tasks. Behaviour: The patient became more socially withdrawn and developed repetitive behaviours, irritability, and a dependency on his wife for daily activities. He later displayed anxiety, repetitive behaviours, and social withdrawal.
Iazzolino et al. [55]	*C9orf72seq*+ (n = 68) *C9orf72seq*- (n = 716)	King’s Stage 1: ALSC9+ (n = 34): Median age at onset is 56.4 years (IQR: 50.5–65.9). Cognitive impairment (ALS-CN; ALSci/ALSbi/ALScbi; ALS-FTD) is 17/13/4. Sex (% female) is 47.1%. ALSC9- (n = 287): Median age at onset is 65.7 years (IQR: 57.6–72.3). Cognitive impairment is 196/76/15. Sex (% female) is 40.1%. King’s Stage 2: ALSC9+ (n = 19): Median age at onset is 58.9 years (IQR: 48.8–65.9). Cognitive impairment is 9/6/4. Sex (% female) is 47.4%. ALSC9- (n = 195): Median age at onset is 66.1 years (IQR: 57.7–72.8). Cognitive impairment is 107/79/9. Sex (% female) is 45.9%. King’s Stage 3: ALSC9+ (n = 15): Median age at onset is 60.4 years (IQR: 54.6–64.9). Cognitive impairment is 4/5/6. Sex (% female) is 66.7%. ALSC9- (n = 191): Median age at onset is 67.8 years (IQR: 57.7–75.7). Cognitive impairment is 97/70/24. Sex (% female) is 44.2%.	Global cognition: ALSC9+ patients consistently had a worse overall cognitive performance across all stages compared to ALSC9-, including lower MMSE scores. Executive Function: ALSC9+ patients showed more severe impairments in executive functions at King’s stage 1 and 3 compared to ALSC9-, with worse scores on tests like FAS, CAT, and TMT B. Verbal Memory: ALSC9+ had poorer verbal memory at King’s stage 1 and 3, as reflected in tests like RAVL-IR and BSRT-IR. Attention and Working Memory: ALSC9+ patients had significant difficulties with attention and working memory at King’s stage 1 and 3, scoring lower on Digit Span tests. Cognitive Flexibility: Cognitive flexibility was notably impaired in ALSC9+ at King’s stage 1 and 3, particularly in TMT B.
Jiskoot et al. [56]	*GRN* (n = 41), *MAPT* = 23; *C9orf72seq* (n = 72); Control (n = 290)	Data on the patients were extracted from the FTD Initiative (GENFI) dataset. All patients had symptomatic FTD. *MAPT*: Mean age of 59.0 years (SD = 7.1). The group comprised 10% females. *GRN*: Mean age of 64.0 years (SD = 8.7). The group comprised 46.3% females. *C9orf72seq*: Mean age of 62.5 years (SD = 7.7). The group comprised 66% females. Controls: Mean age of 46.2 years (SD = 12.9). The group comprised 57.9% females.	Global Cognition: All FTD patient groups showed significantly lower scores on MMSE, CDR plus NACC FTLD-SB, and CBI-R compared to healthy controls. Visuospatial skills (BCFT): Significant differences were found in BCFT Recall and Recognition between all mutation carrier groups (*GRN*, *MAPT*, *C9orf72seq*) and controls, with carriers having significantly lower scores.
Jiskoot et al. [56]	*MAPT* (n = 63), *GRN* (n = 43) or non-carrier group (n = 55).	Longitudinal data of 118 participants from the FTD Risk Cohort of the Erasmus MC University Medical Centre (Rotterdam, the Netherlands). The average age and gender distribution for each group were as follows: phenoconverters 47.9 (SD = 9.3) with 50% females; non-converters 46.7 (SD = 10.6) with 62.3% females; and controls 48.1 (SD = 12) with 50.9% females.	In phenoconverters, decline on the semantic association test (SAT) verbal. Worse performance on Trail Marking Test (TMT-B) and Stroop-III. In *GRN* phenoconverters, decline on the SAT verbal. Worse performance on Stroop-III and a decline on TMT-B. In *MAPT* phenoconverters, decline on the SAT verbal.
Kertesz et al. [57]	*C9orf72seq* (n = 7)	Of the 62 patients tested, 7 patients from 6 different families were found to have expanded repeats in *C9ORF72SEQ*	Patient 1: Pre-diagnosis: Showed significant changes, including psychotic episodes, compulsive eating, hoarding, poor personal hygiene, and aggressive behaviour. His cognitive decline included reduced speech output and impaired personal initiative. At diagnosis (age 58): Exhibited severe language impairment, decreased spontaneous speech, and orofacial movements consistent with tardive dyskinesia. MRI showed extensive parietal and frontal atrophy. Post-diagnosis: Progressed to severe behavioural issues, including inappropriate actions and rigidity in his right arm, suggesting corticobasal degeneration. Patient 2: Pre-diagnosis: Demonstrated detachment, impulsivity, bizarre behaviours, and disinhibition. Notable changes included compulsive eating and wandering behaviour. At diagnosis (age 55): Displayed a vacuous smile, echolalia, and various behavioural symptoms, including impulsivity and restlessness. Neuropsychological testing showed severe apraxia and parkinsonism. Post-diagnosis: Continued to exhibit severe apraxia, rigidity, and dysarthria. His condition declined rapidly, leading to death at age 59. Patient 3: Pre-diagnosis: Experienced word-finding difficulties, social withdrawal, and increased rigidity. Developed compulsive eating and apathy. At diagnosis (age 45): Presented with dysarthria, word-finding difficulties, and significant cognitive impairments. Neuroimaging revealed left frontotemporal atrophy. Post-diagnosis: Deteriorated rapidly with severe disinhibition, apathy, and difficulty with basic activities. Died at age 46. Patient 4: Pre-diagnosis: Showed personality changes, including increased anger and repetitive behaviours, with later onset of word-finding difficulty and choking episodes. At diagnosis (age 59): Presented with severe dysarthria, orofacial apraxia, and significant weakness. MRI indicated frontal and perisylvian atrophy. Post-diagnosis: Diagnosed with FTD/ALS and experienced a rapid decline in physical and cognitive abilities, with death occurring within a year. Patient 5: Pre-diagnosis: Exhibited depressive symptoms, delusions, and childlike behaviours following her husband’s death. Developed compulsive eating and apathy. At diagnosis (age 56): Showed a marked decline in hygiene and social behaviours, with childlike actions and repetitive echolalia. Neuroimaging revealed atrophy in temporal and frontal lobes. Post-diagnosis: Continued to exhibit progressive language decline and incontinence. Resides in a nursing home. Patient 6: Pre-diagnosis: Demonstrated word-finding difficulties, hoarding tendencies, and impulsive purchases. At diagnosis (age 68): Showed mild difficulties with fluency, phonemic and semantic impairments, and obsessive behaviours. MRI revealed mild frontal and temporal atrophy. Post-diagnosis: Became highly routine-bound and emotionally sensitive, with increased obsessive and hoarding behaviours. Patient 7: Pre-diagnosis: Experienced behavioural changes such as disinhibition, impulsivity, and social inappropriateness. Developed visual hallucinations and compulsive behaviours. At diagnosis (age 62): Presented with jovial affect, spastic dysarthria, and significant bulbar symptoms. Neuropsychological testing showed preserved memory with impaired executive functions. Post-diagnosis: Continued to display joviality and progressive bulbar symptoms while remaining active. Hallucinations subsided. Patient 8: Pre-diagnosis: Showed disinhibition, excessive shopping, compulsive eating, and social inappropriateness. Developed visual and auditory hallucinations. At diagnosis (age 63): Exhibited euphoria, apathy, and disorganised behaviour. Neuroimaging indicated severe frontal and temporal atrophy. Post-diagnosis: Developed obsessive behaviours, incontinence, and signs of motor neuron disease, with ongoing visual hallucinations.
Kobayashi et al. [58]	*P301L* (n = q)	A 48-years-old Japanese woman who had frontotemporal dementia with parkinsonism.	Cognitive Function: At her initial neurological examination, she did not exhibit significant memory deficits and was well-oriented, with an MMSE score of 23/30. However, she displayed bradyphrenia (slowness of thought), perseveration, mild emotional incontinence, and mild echolalia. She had slight disorientation but no aphasia or apraxia. Behavioural Changes: Four years after the disease onset, her husband noticed no significant behavioural disorders or apparent dementia. She remained calm and modest, like her elder sister, who had severe dementia and bradykinesia.
Kobayashi et al. [59]	*VCP* (p.Asp395Gly) (n = 1)	A 62-year-old, right-handed Japanese man.	His Mini-Mental State Examination score was 25, failing in the domains of orientation to place, attention/calculations, recall, and repetition. Moreover, his frontal lobe dysfunction was revealed by the Frontal Assessment Battery, a short bedside cognitive and behavioural battery to assess frontal lobe functions (score was 12/18). The results of other neurological examinations were normal.
Koriath et al. [60]	*TBK1* (n = 1)	Case report of a 64 year old with an 18-month history of personality change, including increased rigidity and obsessiveness, apathy, loss of empathy, and development of a sweet tooth.	Global cognition: The patient’s cognitive function was normal, as indicated by perfect scores on the MMSE and strong performance on episodic memory tests. Over time, there was a noticeable decline in cognitive scores, reflecting the progression of his condition. Memory: Decline in episodic memory was evident, with significant impairments in word and face recognition emerging after two years. Language: Naming ability and vocabulary worsened, reflecting progressive language impairment. Calculation: Calculation abilities remained relatively stable, with only a minor decline in the later stages. Visuoperceptual Skills: Performance on visuoperceptual tasks deteriorated, showing severe impairment by the end of the assessment period. Attention and Executive Function: Notable decline was observed in tasks related to attention and executive function, including colour–word interference and trail making.
Kovacs et al. [61]	*SQSTM1* (n = 2)	Two case reports involving men aged 43 and 63 years old.	Case 1 Global Cognition: Mild cognitive impairment, with an MMSE score of 27/30; significant short-term memory issues but intact long-term memory. Language: Reduced expressive language, limited to single words, and mild verbal fluency impairment. Attention: Executive functions, attention, and other cognitive domains were notably affected, with the patient struggling in managing daily tasks and requiring assistance in personal care. Behavioural Assessment: Severe apathy, aggressive behaviour, delusions, and compulsive sharing of personal information. Visuospatial Skills: Problems with handling household items and navigation, indicating potential visuospatial difficulties. Emotion: Marked lack of emotional expression and pervasive apathy. Memory: Severely impaired short-term memory; long-term memory remained intact. Case 2 Global Cognition: Mild cognitive impairment with an MMSE score of 26/30, showing deficits in memory, attention, and executive functions. Language: Minimal issues reported. Behavioural Assessment: Progressive behavioural changes, including disinhibition, impulsivity, paranoia, and inappropriate actions toward strangers. Initial symptoms included carelessness with finances and stubbornness. Emotion: Lack of empathy, increased mistrust, and disinhibition observed.
Laszlo et al. [62]	*C9orf72seq*+ (n = 6) *C9orf72seq*- (n = 12) Healthy control (n = 18)	Patients were recruited through the Scottish Motor Neurone Disease Register. Cognitive function was evaluated using the Edinburgh Cognitive and Behavioural ALS Screen (ECAS), which facilitated the definition of the following groups: Control participants with a mean age of 66 years (SD = 18) and ALS patients with a mean age of 64 years (SEM = 10). Among the ALS cohort, nine patients were classified as having no cognitive impairment (ALSnoci) with a mean age of 66 years (SD = 9), while nine patients were identified with cognitive impairment (ALSci) with a mean age of 62 years (SD = 11). Additionally, the ALS patients were further divided into C9-negative (ALS c9 -ve; n = 12) with a mean age of 58 years (SD = 9) and C9-positive (ALS c9 +ve; n = 6) with a mean age of 67 years (SD = 9). Furthermore, 64% of the control group were male, compared to 56% in the ALS group.	The ECAS total has shown a mean punctuation of 83.2 in the ALS c9 +ve and 107,92 for the ALS c9 −ve. Meanwhile, the ECAS ALS specific has shown an average punctuation of 62.2 in the ALS c9 +ve and 79.5 in the ALS c9 −ve. No analysis was performed on the control groups.
LeBlanc et al. [63]	*C9orf72seq* (n = 1)	A 42-year-old previously healthy male in whom there was identified a repeat expansion of GGGGCC in C9orf72seq, thereby confirming a diagnosis of FTD-ALS.	Disinhibition (e.g., urinating in the kitchen sink at home); and short-term memory impairment. Mood inquiry revealed social withdrawal, irritability, and hypersomnia but no other depressive symptoms (e.g., sadness, guilt, worthlessness). Psychotic symptoms included a seemingly delusional belief that his mother required a specialised ketogenic diet. The patient was noted to be emotional, amotivated, withdrawn, angry, and defensive on assessment. Worsening confusion and cognition (Montreal Cognitive Assessment (MoCA) score 5/30), and signs of regression (e.g., walking around the unit holding a teddy bear).
Lee et al. [64]	*C9orf72seq* (n = 14); Control (n = 290)	All patients had symptomatic FTD, being bvFTD (n = 9) and bvFTD-MND (n = 5). *C9orf72seq*: Mean age of 58.3 years (SD = 7.7). The group comprised 28.6% females. Controls: Mean age of 62.2 years (SD = 4.7). The group comprised 28.6% females.	Global Cognition: Healthy controls scored significantly higher on the MMSE than *C9orf72seq* carriers. Memory: No significant differences were observed between controls and *C9orf72seq* carriers in total learning trials or the 10 min recall of the California Verbal Learning Test. Visuospatial skills (BCFT): There were no significant differences between controls and *C9orf72seq* carriers in the Benson Figure copy score or 10 min recall. Attention: Controls performed significantly better on Digit Span Backward than *C9orf72seq* carriers. Also, controls completed more lines per minute and made fewer errors on Modified Trails compared to *C9orf72seq* carriers. Language (BNT). Healthy controls scored significantly higher than *C9orf72seq*. In fluency tests, controls outperformed *C9orf72seq* carriers on fluency design, producing more correct designs per minute, indicating better visual executive functioning in controls. Controls showed significantly higher scores on letter fluency (‘D’ words) and named more animals per minute than *C9orf72seq*. Calculations: Healthy controls scored higher on calculation tasks compared to *C9orf72seq*. Perceptual Disorders: The NPI composite score for *C9orf72seq* patients was higher than the healthy controls.
Levy et al. [65]	*C9orf72seq* (n = 1)	A 68-year-old man who had bvFTD	Global Cognition: The patient showed mild cognitive impairment with specific deficits in executive function, as suggested by the Trail-Making Test, Part B, and Luria test. Language: No significant language deficits were noted; the patient’s memory and orientation remained intact. Executive Functions: Mild executive dysfunction was indicated by cognitive screening tests, despite normal scores on the MMSE and MoCA. Behaviour: The patient exhibited significant behavioural changes, including rigidity, compulsive and ritualistic behaviours, social withdrawal, and disinhibition. He also displayed stereotypical verbal behaviours and a loss of empathy. Emotion: The patient presented with comorbid manic symptoms, including insomnia, overexcitement, and racing thoughts. His emotional dysregulation required treatment with valproic acid and risperidone.
Mahoney et al. [66]	*MAPT* (n = 22), *GRN* (n = 27), *C9orf72seq* (n = 26)	All patients had symptomatic FTLD. *C9okrf72seq*: Mean age of 55.1 years (SD = 6.6). The group comprised 42% females. *GRN*: Mean age of 57.6 years (SD = 6.5). The group comprised 50% females. *MAPT*: Mean age of 51.7 years (SD = 6.9). The group comprised 41% females.	Global Cognition: Patients with the *C9orf72seq* expansion show widespread cognitive impairments, with significant executive dysfunction affecting all assessed individuals. Memory: Impairments in episodic memory are common, with 87% of patients struggling with recalling personal experiences and visual information. Language: Language difficulties include anomia (word-finding problems) in 47% of patients and issues with effortful speech and agrammatism. Attention: Attention-related problems are suggested by high levels of disinhibition and apathy, affecting focus and task management. Visual and Spatial Processing: Object and space perception remain intact, as all patients assessed showed no impairments in these areas. Emotional Disorders: Apathy and anxiety are prevalent, affecting 49% and 38% of patients initially, respectively, and continuing to be significant at later stages.
Manini et al. [67]	*C9orf72seq* (n = 31) *TARDBP* (n = 10) *FUS* (n = 3) *SOD1* (n = 1)	A total of 972 patients, affected by ALS and other motor neuron diseases (primary lateral sclerosis—PLS and progressive muscular atrophy—PMA) according to the El Escorial revised criteria. The *UNC13A* rs12608932 SNP was genotyped in a cohort of 972 Italian ALS patients, with a prevalence of males (n = 616, 63.4%) over females (n = 356, 36.6%). Only 44 patients (4.8%) had a positive family history for ALS (FALS), while most cases were sporadic (SALS). The C9orf72seq repeat expansion was present in 31 patients (7 FALS, 24 SALS), whereas 10 had a mutation in *TARDBP* (4 FALS, 6 SALS), 3 in *FUS* (all SALS), and 1 in *SOD1* (FALS). Based on the performance at ECAS and according to the Strong revised criteria, 112 out of 254 patients (44.1%) could be classified as ALScn, 45 (17.7%) as ALSbi, 66 (26.0%) as ALSci, and 32 (12.6%) as ALScbi.	Functional and Motor Assessments: ALSFRS-R median score was 40 (IQR: 35–43) in 516 patients. PUMNS had a median score of 9 (IQR: 4–15) among 767 patients. MRC total score was 54 (IQR: 47–58) in 597 patients. LMNS median score was 4 (IQR: 2–6) in 765 patients. Cognitive Assessments: ECAS total score had a median of 105 (IQR: 90–114) among 254 patients. The ALS-specific ECAS score was 78 (IQR: 67–85), and the non-specific score was 27 (IQR: 24–30). Cognitive Phenotypes: Using the Strong revised criteria among 254 patients: 44.1% had ALS with no cognitive or behavioural impairment (ALScn); 17.7% had ALS with behavioural impairment (ALSbi); 26.0% had ALS with cognitive impairment (ALSci); 12.6% had ALS with combined cognitive and behavioural impairment (ALScbi). Frontal Behavioural Inventory (FBI): Total FBI score had a median of 2 (IQR: 0–5) among 203 patients. FBI A score (apathy-related behaviours) was 1 (IQR: 0–4). FBI B score (disinhibition-related behaviours) was 0 (IQR: 0–2).
McDade et al. [68]	*PGRN* (n = 2)	Twins study involving 62-year-old males with a diagnosis of frontotemporal dementia.	Twin 1 (Proband) exhibited severe verbal learning, and memory deficits alongside mild fluent aphasia, characterised by dysnomia and circumlocution. His executive function was average, and his visuospatial abilities remained intact with no impairments detected. Notably, there was a significant discrepancy between his performance IQ and verbal IQ. He experienced difficulties with work performance and daily living activities, which were indicative of cognitive decline related to his diagnosis of frontotemporal dementia. Twin 2 demonstrated deficiencies in learning and memory, with notable forgetfulness in everyday tasks such as paying bills. His language skills were impaired, and there were deficits observed in executive functioning. Despite these challenges, his visuospatial abilities were preserved. Additional symptoms included disinhibition, poor insight, tangential speech, and substantial weight gain, which led to retirement from professional activities due to cognitive difficulties.
Melis et al. [69]	*C9orf72seq* (n = 1)	A 47-year-old male patient was referred to our institution because of uncontrolled seizures, the worsening of previously reported cognitive impairment, and late development of an upper motor neuron syndrome.	Major neurocognitive disorder characterised predominantly by executive dysfunction with attention deficit, poor disease insight, rigidity of thought, difficulty in inhibiting responses with perseverative errors, working memory deficit, verbal memory deficit with florid confabulations, and tangential and bizarre thinking, disinhibition.
Mendez [70]	*GRN* (n = 1)	A 63-year-old right-handed woman who had bvFTD.	Language: Her speech was rapid and tangential but showed normal comprehension and naming abilities. Language tests included word lists, where she named 12 animals/min and nine “F” words/min. Attention and Executive Function: She scored poorly in tests of executive function, including processing speed and mental shifting. Tests like the Digit Span (5 forward, 1 reverse) and the Frontal Assessment Battery (15/18) showed significant impairments. Executive function tests were below the 1st percentile, indicating severe deficits. Memory: Memory was largely preserved. On the Auditory Verbal Learning Test, she recalled 7/10 words at 15 min and recognised all 10. Long-delay free recall was at the 69th percentile. Visuospatial Abilities: She struggled with facial recognition, identifying only 14 of 26 famous faces. However, she performed normally on single-digit and double-digit arithmetic tasks.
Moore et al. [71]	*GRN* (n = 33), *MAPT* (n = 15); *C9orf72seq* (n = 59); Control (n = 248)	Data on the patients were extracted from the FTD Initiative (GENFI) dataset. MAPT mutation carriers, all bvFTD; GRN mutation carriers, 15 bvFTD, 17 PPA, 1 dementia not otherwise specified; and C9orf72seq mutation carriers, 40 bvFTD, 10 FTD with amyotrophic lateral sclerosis, 2 PPA, 1 progressive supranuclear palsy, 3 dementia not otherwise specified. *MAPT*: Mean age of 59.7 years (SD = 6). The group comprised 53% males. *GRN*: Mean age of 63.9 years (SD = 8.4). The group comprised 52% males. *C9orf72seq*: Mean age of 62.2 years (SD = 7.8). The group comprised 66% males. Controls: Mean age of 46.5 years (SD = 13). The group comprised 42% males.	Global Cognition: Healthy controls scored significantly higher than *C9orf72seq* carriers on the FTLD-CDR. Language (modified Camel and Cactus Test): All three symptomatic mutation carrier groups scored significantly lower than controls, regardless of the disease stage.
Nelson et al. [72]	*GRN* (n = 47), *MAPT* (n = 21); *C9orf72seq* (n = 67); Control (n = 267)	Data on the patients were extracted from the GENFI dataset. All patients had symptomatic FTD. *C9orf72seq*: Mean age of 62.6 years (SD = 9.4). The group comprised 66% males. *GRN*: Mean age of 63.0 years (SD = 7.4). The group comprised 47% males. *MAPT*: Mean age of 58.9 years (SD = 9.4). The group comprised 57% males. Healthy Controls: Mean age of 46.4 years (SD = 13.0). The group comprised 41% males.	Global Cognition: All FTD patient groups showed significantly lower scores on the MMSE and CBI-R compared to healthy controls.
Ogonowski et al. [73]	*TREM2* (n = 2) Control (HC, n = 2)	Two patients from unrelated Colombian families and the third case from the USA of Mexican origin with heterozygous *TREM2* exon 3 variants (c.469C>T, p.H157Y, rs2234255). The two Colombian cases exhibited a bvFTD clinical profile, and the third case presented with motor neuron disease (MND) with age-matched, sex-matched and education-matched groups—a healthy control group (HC) and a group with FTD with neither *TREM2* mutations nor family antecedents (Ng- FTD and Ng-FTD-MND).	General Cognitive State: In the MoCA, Case 1 and Case 2 exhibited significantly lower scores than both HCs and the Ng- FTD group. Additionally, Ng- FTD presented significantly lower scores than HC in MoCA scores. No differences when compared with Ng- FTD- MND nor with the Ng- FTD group were observed. Executive Functions: Both *TREM2* cases showed lower scores on total IFS (Ineco Frontal Screening) than HC and the Ng- FTD group. In addition, *TREM2* cases exhibited lower Hayling scores than HC. Also, Case 3 showed lower scores on the total of correct trials in the Stroop task compared with Ng- FTD- MND cases. Behavioural changes: *TREM2* cases showed significantly higher scores in total FrSBe (Frontal Systems Behavioural Scale) than HC. Case 2 exhibited lower total FrSBe scores than the Ng- FTD group. Individualised analyses on each FrSBe subfactor revealed higher scores for *TREM2* cases compared with HC. Moreover, Case 3 showed significantly higher scores on the total scores of the NPI than Ng- FTD- MND cases. The patient exhibited worst scores on agitation, apathy, disinhibition, motor problems, sleep disturbances, and eating habits. Social Cognition: Both *TREM2* cases attained significantly lower scores for social cognition (RMET) compared with HC. Case 3 showed significantly higher scores on the items tracking social norms (NPI disinhibition score) than Ng- FTD- MND cases.
Olney et al. [74]	*C9orf72seq*, *MAPT*, *GRN*	2mFTLD-CDR 5 0 (mild FTLD with CDR 5 0): Mean age of 47.53 years (95% CI = 44.96 to 50.1). The group comprises 43.1% females. 1mFTLD-CDR 5 0 (moderate FTLD with CDR 5 0): Mean age of 43.95 years (95% CI = 41.22 to 46.68). The group comprises 47.2% females. 1mFTLD-CDR 5 0.5 (moderate FTLD with CDR 5 0.5): Mean age of 55.44 years (95% CI = 52.03 to 58.85). The group comprises 50.0% females. 1mFTLD-CDR 5 1 (moderate FTLD with CDR 5 1): Mean age of 60.17 years (95% CI = 57.96 to 62.38). The group comprises 41.7% females.	*MAPT*: The MoCA and MINT were commonly abnormal across all severity levels. Consistent abnormalities were noted, with MINT often being the most or second most common abnormal test. *GRN*: Common abnormalities included the Craft story recall task, the Trail Making Test, and “F” word fluency. The MoCA was also frequently abnormal but less so compared to MAPT. *C9orf72seq*: The MoCA was commonly abnormal, but there was less consistency across severity levels compared to MAPT and GRN. The MINT was the most common abnormal test in the 1m FTLD-CDR 5 0 group, but many individuals showed abnormalities on different tests.
Olszewska et al. [75]	*SCA17* (n = 1)	A 44-year-old female diagnosed with a complex familial FTD with cerebellar features and co-inheritance of an *MAPT* variant and *SCA17* CAG/CAA expanded repeat	Global Cognition: The patient experienced severe cognitive decline, with significant impairments in executive functions and problem-solving. Memory: Memory issues included poor working and episodic memory, with better preservation in delayed recall but notable difficulty with immediate recall. Language: Language was marked by difficulty finding words, effortful speech, and reduced elaboration, though reading comprehension and single-word repetition were intact. Attention and Executive Function: Severe deficits in attention and executive functions were observed, including poor task perseverance, mental control, and cognitive flexibility. Emotional Disorders: The patient showed emotional distress and apathy but did not exhibit pervasive depression. Visuospatial Skills: Visuospatial abilities were impaired, especially in complex figure reproduction, while basic visual perception was preserved.
Peakman et al. [76]	*MAPT* (n = 77), *GRN* (n = 187), *C9orf72seq* (n = 193) Control (n = 268)	Data on the patients were extracted from the GENFI dataset. All patients had symptomatic FTD. *C9orf72seq*: Mean age of 51.5 years (SD = 13.8). The group comprised 49.2% males. *GRN*: Mean age of 50.8 years (SD = 13.6). The group comprised 39.6% males. *MAPT*: Mean age of 45.5 years (SD = 13.8). The group comprised 45.5% males. Non-carrier controls: Mean age of 46.4 years (SD = 12.9). The group comprised 41.4% males.	Global Cognition: FRS scores decrease and CDR+NACC-FTLD-SB scores increase with disease progression in FTD, showing a strong correlation in *GRN*, *C9orf72seq*, and *MAPT* mutation carriers.
Pengo et al. [77]	*GRN* (n = 66) *C9orf72seq* (n = 26) *MAPT* (n = 3)	All patients were consecutively recruited at the Centre for Neurodegenerative Disorders, Department of Clinical and Experimental Sciences, University of Brescia, Italy, from July 2007 to July 2021. A total of 531 FTD patients were consecutively recruited, namely 345 patients with bvFTD, 118 with the agrammatic variant of primary progressive aphasia (avPPA), and 68 with the semantic variant (svPPA). The study group consisted of 258 women (mean age 66.4 ± 8.1 years old) and 273 men (mean age 65.4 ± 8.4 years old). The main finding of the study is that the bvFTD phenotype was more common in men (74%), whereas PPA was more common in women (57%). The average years of education was 9.1 (SD = 4.3).	Global disease severity measured by the DR Dementia Staging Instrument, plus behaviour and language domains from the National Alzheimer’s Coordinating Centre and Frontotemporal lobar degeneration modules—sum of boxes (CDR plus NACC FTLD—SOB) showed that men presented more frequent behavioural disturbances, whereas women had a higher cognitive impairment when considering specific tasks. Behavioural disturbances were rated by the Italian version of the Frontal Behavioural Inventory (FBI) showed that men exhibited more personality/behavioural symptoms. In particular, looking at FBI subitems, men presented more severe apathy, irritability, poor judgement, aggressivity, and hypersexuality. Women reached lower scores, corrected for disease stage, in the Trail Making Test parts A and B, semantic fluency, Short Story Recall Test, and Rey Complex Figure, copy.
Pletnikova et al. [78]	C9orf72seq (n = 3)	3 women between 66.6 and 71 years old. They had an initial diagnosis of Alzheimer’s disease.	In the first three years of illness, symptoms primarily included amnesia, with some showing irritability/agitation, dissocial behaviour, or neglect of self-care. Disorientation, executive dysfunction, aphasia, agnosia, and apraxia were uncommon at onset but became present later. All carriers developed depression, irritability, or agitation over time. Motor dysfunction, including tremors, rigidity, and an abnormal gait, emerged after the fifth year, with no cerebellar signs observed. Hyperphagia was absent initially, but anorexia, hypophagia, and dysphagia developed later. Incontinence also appeared late.
Poos et al. [79]	*MAPT* (n = 29), *GRN* (n = 20), *C9orf72seq* (n = 31) Control (n = 24)	Patients had a clinical diagnosis of bvFTD and underwent one or multiple neuropsychological assessments. *C9orf72seq*: Mean age of 62.1 years (SD = 9.1). The group comprised 58.1% males. *GRN*: Mean age of 60.4 years (SD = 7.4). The group comprised 42.9% males. *MAPT*: Mean age of 52.6 years (SD = 5.5). The group comprised 65.5% males. Non-carriers: Mean age of 56.1 years (SD = 5.7). The group comprised 54.2% males.	*C9orf72seq* Mutation Carriers: *C9orf72seq* mutation carriers exhibit significant impairments in executive functioning and language, with SCWT I and II being particularly sensitive for executive dysfunction. They also show notable deficits in attention and processing speed. However, there were no significant impairments observed in memory, visuoconstruction (Clock Drawing), or working memory (WAIS-III Digit Span). *MAPT*: MAPT mutation carriers present significant impairments in executive functioning, language, and attention/processing speed. The RBMT direct and delayed recall tests are particularly sensitive for language deficits, while the RAVLT immediate and delayed recall tests highlight attention and processing speed issues. Additionally, they show significant impairments in both immediate and delayed memory recall. No significant issues are noted in visuoconstruction (Clock Drawing) or working memory (WAIS-III Digit Span). *GRN*: *GRN* mutation carriers show significant declines in global cognition, as evidenced by lower scores on the MMSE and FAB. They are most notably impaired in executive functioning, with the WCST being the most sensitive test for identifying these deficits. Impairments are also evident in language and attention/processing speed. In terms of memory, *GRN* carriers have significant impairments in immediate recall and specific deficits in the VAT. There are no significant issues in visuoconstruction (Clock Drawing) or working memory (WAIS-III Digit Span).
Poos et al. [80]	*MAPT* (n = 24), *GRN* (n = 46), *C9orf72seq* (n = 66) Control (n = 255)	Data on the patients were extracted from the GENFI dataset. Pathogenic variant carriers met diagnostic criteria for bvFTD, PPA, and FTD-ALS. *C9orf72seq*: Mean age of 62.2 years (SD = 8.9). The group comprised 36.4% females. *GRN*: Mean age of 63.6 years (SD = 7.9). The group comprised 50% females. *MAPT*: Mean age of 57.3 years (SD = 10.2). The group comprised 33.3% females. Non-carrier controls: Mean age of 45.3 years (SD = 12.8). The group comprised 56.9% females.	*C9orf72seq*: Despite an initial lower performance, cognitive decline was relatively stable over time, with minimal progression compared to other genetic groups. *MAPT*: Initial cognitive deficits in memory progressed to a more widespread decline in language, attention, executive function, and social cognition as the disease advanced. *GRN*: Significant initial impairment in all cognitive domains was observed, with the most rapid decline in verbal fluency, language, and social cognition during the symptomatic stage.
Russell et al. [81]	*MAPT* (n = 18), *GRN* (n = 32), *C9orf72seq* (n = 53) Control (n = 246)	Data on the patients were extracted from the GENFI dataset. Pathogenic variant carriers met diagnostic criteria for bvFTD, PPA, and FTD-ALS. *C9orf72seq*: Mean age of 62.3 years (SD = 8.0). The group comprised 36.2% females. *GRN*: Mean age of 64.2 years (SD = 8.4). The group comprised 47.2% females. *MAPT*: Mean age of 59.8 years (SD = 6.0). The group comprised 40.0% females. Healthy Controls: Mean age of 46.0 years (SD = 12.8). The group comprised 58.0% females.	Global Cognition: All patient groups showed significantly lower scores on MMSE and FTLD-CDR-SOB compared to controls.
Samra et al. [82]		Data on the patients were extracted from the GENFI dataset. Pathogenic variant carriers met diagnostic criteria for bvFTD, *C9orf72seq*: Mean age of 67.2 years (SD = 7.4). The group comprised 56% females. *GRN*: Mean age of 62.3 years (SD = 7.9). The group comprised 56% females. *MAPT*: Mean age of 59.1 years (SD = 7.7). The group comprised 20% females. Controls: Mean age of 60.2 years (SD = 7.1). The group comprised 55% females.	Global Cognition: All patient groups showed significantly lower scores on MMSE and FTLD-CDR compared to controls. *C9orf72seq*: Significantly impaired on the Progressive Aphasia Severity Scale (PASS) with notable difficulties involving decreased fluency, impaired word retrieval, and impaired sentence comprehension. They also had the lowest scores on the Boston Naming Test and category fluency compared to other groups. *GRN*: Showed substantial deficits on the PASS, particularly in decreased fluency, impaired word retrieval, and impaired functional communication. Their performance on the Boston Naming Test and category fluency was worse than controls but not as severely impaired as C9orf72seq carriers. *MAPT*: Exhibited severe impairments on the PASS, including decreased fluency, impaired word retrieval, and impaired sentence comprehension. They performed the poorest on the BNT and category fluency among the groups.
Samra et al. [83]	*MAPT* (n = 25), *GRN* (n = 52), *C9orf72seq* (n = 72) Control (n = 310)	Data on the patients were extracted from the GENFI dataset. All patients had symptomatic FTD. *C9orf72seq*: Mean age of 62.7 years (SD = 9.3). The group comprised 41% females. *GRN*: Mean age of 63.5 years (SD = 7.7). The group comprised 46% females. *MAPT*: Mean age of 57.0 years (SD = 10.1). The group comprised 36% females. Controls: Mean age of 46.0 years (SD = 12.7). The group comprises 56% females.	Global Cognition: All patient groups showed significantly lower scores on MMSE and FTLD-CDR compared to controls. *C9orf72seq*: In symptomatic C9orf72seq mutation carriers, neuropsychiatric symptoms were highly prevalent, with frequent anxiety, depression, and hallucinations. *GRN*: Symptomatic *GRN* carriers also had common neuropsychiatric symptoms, notably anxiety and depression, but with less frequent hallucinations compared to C9orf72seq carriers. *MAPT*: Symptomatic *MAPT* carriers experienced anxiety, depression, and notable changes in humour. Hallucinations were less common and less severe compared to *C9orf72seq* and *GRN* carriers.
Santamaría-García et al. [84]	*TREM2* (n = 1) *TARDBP* (n = 1) *MAPT* (n = 1) *MAPT H1H2* (n = 3) *MAPT H2H2* (n = 3) Healthy controls (n = 10)	Forty-two FTD patients who fulfilled the revised criteria for probable bvFTD. One patient (2.4% of the total sample) carried a missense variant of MAPT (c.454G > A, p.Ala152Thr, rs143624519), one patient (2.4% of the sample) had a missense variant in the TARDBP gene (c.1147A > G, p.I383V, rs80356740), and one patient (2.4% of the sample) exhibited a missense variant of *TREM2* (c.140G > A, p.Arg47His, rs75932628). Six patients (14.2% of the sample) had *H1H2* and *H2H2 MAPT* genotypes. Among these patients, three patients presented with the *H2H2* and three patients presented with the *H1H2* genotype. Ten patients with sporadic presentation did not exhibit particular mutations and showed a combination of *H1H1* tau genotype and ε3ε3 variants of APOE. The *MAPT* gene group (GR1) had an average age of 66 years and included only one male participant with no females. In contrast, the *TARDBP* gene group (GR1) had a considerably younger average age of 48 years and similarly consisted of one male participant with no females. The *TREM2* gene group (GR1) was slightly older, with an average age of 63 years, and was exclusively composed of one female participant. The *Tau Haplotypes* (GR2) group had an average age of 66.99 years (SD = 5.71) and included an equal number of males and females, with six participants of each gender. The *APOE Haplotypes* (GR2) group was older, with an average age of 70.01 years (SD = 8.76), and also consisted of six males and six females. Lastly, the S-FTD (GR3) group had an average age of 68.29 years (SD = 9.22) and featured a balanced gender distribution of five males and five females.	Cognitive Assessment: The TARDBP gene group showed the lowest scores across several cognitive domains, including the Montreal Cognitive Assessment (MoCA) and Rey–Osterrieth Figure tasks, indicating greater cognitive impairment compared to other groups. The S-FTD group (GR3) generally performed better in cognitive assessments, with higher scores on MoCA and Rey–Osterrieth Figure compared to other genetic groups. Executive functioning was most impaired in the *TREM2* gene group, which had the lowest scores on the Ineco Frontal Screening and verbal inhibitory task. Social Cognition: The Reading Mind in the Eyes and Reading Mind in the Faces tasks showed slight variations across groups, with the S-FTD group (GR3) performing slightly better than others in these social cognition tasks. Neuropsychiatric Symptoms: Chronic symptoms (apathy, disinhibition, and disorganised behaviour) were generally highest in the TREM2 gene group, particularly in chronic disorganised behaviour. Current neuropsychiatric symptoms were more severe in the TARDBP gene group, particularly in apathy and disorganised behaviour, compared to other groups.
Sassi et al. [85]	*GRN* (n = 3), *C9orf72seq* (n = 1), *CSF1R* (n = 1), *SORL1* (n = 1), *CLCN6* (n = 1	The clinical subtypes within the cohort were predominantly bvFTD, which accounted for 75% of the cases, followed by PPA at 21%. A small proportion (1%) of patients exhibited FTD with amyotrophic lateral sclerosis (FTD-ALS). The average age of onset for the disease was 63 years, ranging from 43 to 85 years. A significant portion of the cohort, 55%, had early-onset FTD (before 65 years of age), and 24% experienced very early-onset FTD (before 55 years of age).	Memory: Memory deficits were noted, particularly in relation to the mutation in *CLCN6*, which is associated with FTD with memory onset in some family members. Language: Significant language difficulties were observed, including issues with comprehension and expression. These impairments were prominent in patients with mutations in *SORL1*, which were linked to language deterioration and primary progressive aphasia (PPA). Behavioural symptoms: Mutations in *CSF1R* and *CLCN6* were linked to FTD, manifesting in behavioural symptoms such as personality changes, disinhibition, and repetitive behaviours.
Saxon et al. [13]	*C9orf72seq* (n = 13)	The sample consists of patients diagnosed with bvFTD (n = 6) and FTD-ALS (n = 7), with a mean age of 59.5 years (SD = 7.5).	ALS patients with the *C9ORF72SEQ* expansion exhibited specific cognitive impairments, particularly in language, such us spelling (PALPA Spelling Test), sentence comprehension (PALPA Sentence Comprehension Test), and block sorting (DKEFS Block Sorting Task), with additional trends suggesting difficulties in sentence ordering (Sentence Ordering Task), semantic association (Pyramids and Palm Trees Test), and category fluency (Category Fluency Index). However, these patients did not show significant differences in behavioural measures or overall illness severity compared to those without the expansion.
Scarioni et al. [86]	*TARDBP* (n = 46) *FUS* (n = 7)	Eighty-seven brain donors from The Netherlands Brain Bank cohort (2008–2017) diagnosed with FTLD were included: 46 FTLD-TAR DNA-binding protein 43 (FTLD-TDP), 34 FTLD-tau, and seven FTLD-fused-in-sarcoma (FTLD-FUS). FTLD-*TDP*. Age = 59.4 years. The mean age at death for FTLD-TDP donors was 67.4 years, reflecting a slightly longer disease duration compared to the FTLD-FUS group. FTLD-*tau*. Age = 56.9 years. The average age at death for FTLD-tau donors was 66.1 years. FTLD-*FUS*. Age = 48.6 years. The average age at death was 56.1 years.	In the FTLD-*TDP* group (46 donors), depression was present in 13%, which is significantly lower compared to the FTLD-*FUS* group. Mania was reported in 6.5% of FTLD-TDP donors. A notable 23.9% of FTLD-TDP donors experienced hallucinations, which is significantly higher than in the FTLD-tau and FTLD-FUS groups. The rate of delusions was 8.7%. High levels of disinhibition (71.7%) and apathy (67.4%) were observed, with median scores for these symptoms being consistent with the overall sample. Perseveration/compulsion and hyperorality were noted in 58.7% and 56.5% of donors, respectively. FTLD-*tau* Group: Among the 34 FTLD-*tau* donors, 20.6% experienced depression, similar to the overall sample. Mania was absent in this group. Hallucinations were not reported, and delusions were present in 11.8%, which was relatively high but not statistically significant compared to other groups. Disinhibition was observed in 76.5% of FTLD-tau donors, slightly higher than the overall average, and apathy was also high at 76.5%. Perseveration/compulsion and hyperorality were reported in 73.5% and 76.5% of FTLD-tau donors, respectively, with median scores comparable to the overall sample. FTLD-*FUS* Group: In the smallest group, FTLD-*FUS* (7 donors), a high prevalence of depression (71.4%) was noted, significantly higher than in the FTLD-TDP and FTLD-tau groups. Mania was reported in 14.3% of FTLD-FUS donors. Hallucinations and delusions were not observed in this group. Disinhibition was present in 85.7% of FTLD-FUS donors, which is significantly higher compared to the other groups, and apathy was also high at 85.7%. Perseveration/compulsion and hyperorality were reported in 71.4% of donors, with the median scores being similar to or slightly higher than the overall sample.
Schiava et al. [87]	*VCP* (n = 234)	*VCP* (n = 234). Seventy per cent (163/234) were males, and the mean age at last assessment was 56.8 (SD = 9.6) years.	Cognitive impairment was identified in 25.5% (59/231) of the patients, of which frontotemporal dementia (FTD) was the most frequent pattern (33/59) followed by a mixed cognitive impairment (25/59).
Seelaar et al. [88]	*TDP* (n = 19), *MAPT* (n = 10), control (n = 10)	Familial FTLD-*TDP*: Mean age at onset of 56.9 years (SD = 8.9). The group comprises 44.4% females. *MAPT*: Mean age at onset of 49.8 years (SD = 5.9). The group comprises 50% females. Controls: Not applicable, as there is no age at onset data for this group in the context provided. The group comprises 40% females.	*MAPT*: On the BNT, MAPT mutation patients performed significantly worse compared to familial FTLD-TDP patients. No differences were found in other cognitive domains between these two patient groups. *TDP*. Patients with FTLD-TDP exhibited significant memory impairment compared to controls (*p* = 0.011), but no notable differences were found in other cognitive domains when compared to controls.
Sellami et al. [89]	*GRN* (n = 75), *C9orf72seq* (n = 60), *MAPT* (n = 32)	*GRN*: Mean age is 51.3 years (SD = 11.4). The group comprises 64% females. Symptomatic: 16%. Presymptomatic: 84%. *C9orf72seq*: Mean age is 52.1 years (SD = 14.2). The group comprises 51.7% females. Symptomatic: 38%. Presymptomatic: 62%. *MAPT*: Mean age is 44.3 years (SD = 12). The group comprises 50% females. Symptomatic: 31%. Presymptomatic: 68%.	Global cognition: The *C9orf72seq* group, in particular, has a higher proportion of severely impaired individuals. Hallucinations: Visual, auditory, and tactile hallucinations were reported similarly across the *GRN*, *C9orf72seq*, and *MAPT* genetic groups. The severity of these hallucinations was generally mild for all groups. Delusions: Delusions were observed in a small proportion of participants in each genetic group, with no significant differences in severity between them. Depression: The incidence of depression was similar across all genetic groups, with comparable levels of severity. Anxiety: Anxiety was present in a similar proportion of participants in each genetic group, and the severity was comparable across groups.
Shinagawa et al. [90]	*C9orf72seq* (n = 4)	Four case reports. All of them are men who died at 58, 59, 64, 70 years. All 4 had unclassifiable FTLD with TAR DNA-binding protein inclusions.	Global Cognition: All cases exhibit progressive deterioration, with variations in severity and affected areas. Language: Notable language deficits are present in all cases, with differences in severity and types of impairment. Visuoperception: Generally preserved in most cases. Executive Functions: Severely affected in all cases, impacting planning, decision-making, and cognitive flexibility. Attention: Problems with concentration and distractibility, with variations in severity. Behaviour: Inappropriate behaviours, apathy, and changes in social and personal conduct. Emotion: Changes in emotionality and empathy, with the presence of delusions and hallucinations in some cases.
Silva-Spinola et al. [91]	*GRN* (n = 20) *C9orf72seq* (n = 13) Sporadic-FTD (n = 30)	Patients were recruited from the memory clinic of the Neurology Department of Centro Hospitalar e Universitário de Coimbra. All genetic cases with available CSF samples were included (20 with GRN mutations [GRN-FTD] and 13 with an hexanucleotide repeat expansion in C9orf72seq gene [C9orf72seq-FTD]) and compared with 30 FTD cases with no mutations (sporadic FTD). *GRN*-FTD. 20 participants, 9 males and 11 females), the mean age was 57.2 years. *C9orf72seq*-FTD. 13 participants, 7 males and 6 females, the mean age was 63.0 years. Healthy control. 37 participants, 18 males and 19 females, they had a mean age of 60.1 years.	In the Sporadic-FTD group, cognitive performance shows a broad range of deficits. On the MMSE, a measure of general cognitive function, scores are significantly lower than expected, reflecting notable cognitive impairment. The MoCA scores also indicate reduced cognitive performance, with deficits across various domains. Tasks such as the Cancellation Task, Digit Span, and Semantic Fluency show mild to moderate impairments. Performance on executive functions and memory tasks like the Verbal Paired-Associate Learning and Logical Memory (Immediate Recall) reflect challenges in these areas, suggesting difficulties with attention, working memory, and executive control. Basic written calculations and visual memory tests show relatively less impairment, indicating that some cognitive functions are relatively preserved. The *GRN*-FTD group exhibits more pronounced cognitive deficits compared to the Sporadic-FTD group. On the MMSE, individuals show considerable impairment, which is consistent with their performance on the MoCA. Key neuropsychological tests reveal severe deficits, particularly in areas such as Digit Span and Semantic Fluency, reflecting significant challenges in attention, working memory, and language. The Cancellation Task and Graphomotor Initiative also indicate notable impairments, suggesting difficulties with both executive functions and motor control. Memory assessments, including the Logical Memory (Immediate Recall) and Autobiographic Memory, show substantial deficits, highlighting significant challenges in both immediate and long-term memory. Basic written calculations are notably impaired in this group, suggesting broader cognitive dysfunction affecting daily tasks. In the *C9orf72seq*-FTD group, cognitive performance shows notable impairments across various domains. The MMSE and MoCA scores indicate significant overall cognitive deficits. The group demonstrates marked difficulties in tasks such as the Cancellation Task and Semantic Fluency, similar to the GRN-FTD group. Executive function tasks, such as the RPCM-ab Series and Naming, show substantial deficits, reflecting challenges in cognitive flexibility and language. Memory tasks, including the Verbal Paired-Associate Learning and Logical Memory (Delayed Recall), highlight difficulties with both immediate and delayed recall, indicating pronounced memory impairment. Performance on basic written calculations and visual memory tests show significant challenges, reflecting widespread cognitive dysfunction in this cohort.
Snowden et al. [92]	*PGRN* (n = 3)	Three case reports were identified: the proband, her sister, and another proband from another family who died at 71, 75, and 66 years old, respectively. All al them were diagnosed with FTDL.	Patient 1: Exhibited language difficulties with hesitant speech and phonemic errors, though grammar and comprehension were intact. She had mild executive function impairments, including poor sequencing and idea generation, with an uneven memory, particularly in verbal tasks. Spatial and perceptual functions were intact, and she performed well in calculations and praxis. Patient 2: Struggled primarily with language expression, especially naming, with empty speech and word retrieval issues. Despite poor verbal fluency, her comprehension remained strong. Memory was impaired for verbal material but intact for visual tasks. She performed well in executive tasks that did not require verbal input, with preserved visual perception and praxis. Patient 3: Showed severe dysexecutive syndrome, with preserved language, perception, and spatial skills. Speech was economical but free from errors. Memory was weak for free recall but improved with cues. Executive function was severely impaired, marked by poor sorting, sequencing, and verbal fluency, indicating frontal lobe dysfunction.
Snowden et al. [93]	*FUS* (n = 5)	Five case reports, with ages ranging from 22 to 52 years, including 20% female patients, were diagnosed with Frontotemporal Lobar Degeneration with *FUS* Pathology (n = 4) and Neurodegenerative Frontotemporal Dementia with *FUS* Pathology (n = 1).	aFTLD-U is characterised primarily by executive function impairments, such as difficulties with planning, shifting tasks, and perseveration. Patients exhibit a slowed initiation of tasks, often staring blankly and struggling to engage, indicative of subcortical–frontal dysfunction. Notably, aFTLD-U does not present with prominent cortical symptoms like aphasia, agnosia, spatial disorientation, or apraxia. Memory performance is inefficient but consistent with the observed executive impairments. NIFID presents with significant memory loss along with aphasia and apraxia, often starting at an older age compared to aFTLD-U. This form of dementia involves pronounced difficulties with language and motor coordination. Unlike aFTLD-U, patients with NIFID may show clear cortical symptoms and initially might be misdiagnosed with Alzheimer’s disease due to the memory impairment and motor symptoms.
Solje et al. [94]	*C9orf72seq* (n = 36)	36 patients carrying the *C9orf72seq* expansion and suffering from bvFTD (N = 32) or from bvFTD with motor neuron disease (bvFTD-MND, N = 4). Age at the onset of symptoms: Mean age of 59.3 years (SD = 6.6). The range is 44 to 76 years.	Global Cognition: All patients exhibited symptoms of frontotemporal lobar degeneration (FTLD) at diagnosis. Visuoperception: Typically preserved. Executive Functions: Significantly impaired in bvFTD, with a greater number of criteria met in patients with pure bvFTD compared to those with bvFTD-MND. Behaviour: Notable behavioural changes, including apathy and disinhibition. Fewer behavioural changes in bvFTD-MND patients. Emotion: Emotional changes present, including difficulties with emotional regulation.
Spinelli et al. [95]	*TARDBP* (n = 11); Controls (n = 22)	*TARDBP* (TAR DNA-binding protein 43): Mean age of 59.78 years (SD = 8.17). The group comprises 45.5% females. Healthy Controls: Mean age of 58.99 years (SD = 6.08). The group comprises 50% females.	Global Cognition: *TARDBP* patients have similar MMSE scores to healthy controls. Memory: No notable differences in Digit Span Forward, delayed recall, or recognition tasks compared to controls. Executive Functions: Comparable performance to controls on PM (Coloured Progressive Matrices) and Digit Span Backward tests. Visuospatial Abilities: Scores on the Rey Figure copy test are similar to those of controls. Language (BADA): Action Naming: *TARDBP* patients show a lower performance compared to controls. Noun Naming: Performance is poorer compared to controls, though not significantly different. Fluency: No significant differences in verbal fluency compared to controls.
Staffaroni et al. [96]	*C9orf72seq* (n = 347) *GRN* (n = 281) *MAPT* (n = 168) Non-carriers (n = 412)	Participants were enrolled through Advancing Research and Treatment for Frontotemporal Lobar Degeneration (ARTFL; NCT02365922) and Longitudinal Evaluation of Familial Frontotemporal Dementia Subjects (LEFFTDS; NCT02372773), which recently combined into the ARTFL/LEFFTDS Longitudinal Frontotemporal Lobar Degeneration (ALLFTD; NCT04363684) study. Most symptomatic participants presented with behavioural variant *FTD* (bvFTD, 68.6%), followed by primary progressive aphasia (PPA, 12.7%), which was driven largely by *GRN* (33.8% of symptomatic *GRN*). The average number of visits per mutation carrier was 2.1 (SD = 1.1).	*GRN* exhibited the most rapid CDR^®^+NACCFTLD-SB changes following symptom onset. *C9orf72seq* expansion carriers performed worse than controls on Trails A and B at all DA epochs (Table S5 [96]). GRN performed worse than controls on Trail A at all epochs, and worse than controls on Trail B in the −10 to 0 epoch. *MAPT* mutation carriers exhibited impairments in the Figure Copy in the −10 to 0 epoch, with a trend towards impairment on the Multilingual Naming Test (MINT) in this epoch. Longitudinally, the most rapid change in the symptomatic stage relative to controls was observed for Trails A and B in C9orf72seq, Trail A, MINT, and Benson Copy in *GRN* and the MINT and Trail B in *MAPT*
Tan et al. [97]	*UNC13A* (n = 2216)	All patients have ALS. *UNC13A* Genotype A/A (n = 854): Mean age at onset is 63.5 years (median, IQR: 55.6–70.3), and the group comprises 40.6% females. *UNC13A* Genotype A/C (n = 988): Mean age at onset is 65.6 years (median, IQR: 59.0–71.8), and the group comprises 39.2% females. *UNC13A* Genotype C/C (n = 374): Mean age at onset is 65.5 years (median, IQR: 58.9–71.4), and the group comprises 42.2% females.	Global Cognition: The C-allele of UNC13A was associated with lower overall ALS-specific scores on the Edinburgh Cognitive and Behavioural ALS Screen (ECAS). This indicates a generally poorer cognitive performance in C-allele carriers, although the proportion of patients scoring below cutoffs did not significantly differ by genotype. Language: Patients with the C-allele scored lower in the language domain of the ECAS. This reflects more difficulty in language tasks, as measured by language-specific tests within the ECAS. Verbal fluency, assessed by tests such as FAS, did not show significant differences between genotypes. Executive Function: The C-allele was linked to lower scores in executive function tests, including the Verbal Fluency Test (FAS), the Cognitive Assessment Tool (CAT), and the TMT B-A. This indicates impaired cognitive flexibility and planning abilities. Visual and Spatial Functions: No significant differences were found in visual and spatial function scores between genotypes, as assessed by visual and spatial tasks within the ECAS. Behaviour: The C-allele was associated with higher frequencies of behavioural impairments, such as disinhibition, and higher scores on the ALS-FTD Questionnaire (ALS-FTD-Q). This suggests an increased likelihood of meeting criteria for ALS with behavioural impairment (ALS-bi) and frontotemporal dementia (ALS-FTD). Memory: Memory performance, assessed by tasks such as recall and recognition on the ECAS, did not show significant differences across genotypes. Memory functions appeared relatively preserved regardless of UNC13A genotype. Attention and Working Memory: The C-allele was linked to poorer performance in attention and working memory tasks, including Digit Span Forward (FW) and Backward (BW), as measured by the ECAS.
Tang-Wai et al. [98]	*PSEN1* (n = 1)	A 59-years-old woman who had familial FTD.	Global Functioning/Screening: The proband’s global cognitive function, as measured by the Short Test of Mental Status (STMS) and the Mattis Dementia Rating Scale, significantly declined from mild impairment at age 57 to severe dementia by age 59. Attention/Concentration: Attention and concentration, assessed by the Trail-Making Test and Stroop Test, showed a deteriorating performance, with increased completion times and an impaired ability to manage conflicting information. Language Functioning: Language assessments, including the Boston Naming Test and COWAT, indicated worsening difficulties in naming, verbal fluency, and language retrieval over time. Planning, Reasoning, Problem-Solving: The Wisconsin Card Sorting Test revealed a decrease in the ability to shift cognitive strategies and complete tasks, indicating impaired problem-solving and cognitive flexibility. Memory and Learning: Memory tests, such as the Wechsler Memory Scale—Revised and Free and Cued Selective Reminding Test, showed severe impairments in both verbal and visual memory, with significant declines in recall abilities. Visuoconstructive and Visuospatial Skills: Visuoconstructive abilities, evaluated by the Rey–Osterreith Complex Figure, and visuospatial skills, assessed by Visual Form Discrimination, showed a marked deterioration, while line orientation judgement remained intact.
Temp et al. [99]	*SOD1* (n = 4), *C9orf72seq* (n = 2)	Eighty-three amyotrophic lateral sclerosis (ALS) patients and their family members were prospectively recruited at outpatient clinics in Rostock and Magdeburg, Germany. The study employed the Strong criteria for profiling ALS and Frontotemporal Dementia Spectrum Disorders (ALS-FTSDs), allowing for classification into the following subgroups: ALS without cognitive impairment (ALSni) with a mean age of 60.25 years (SD = 11.74), ALS with cognitive impairment (ALSci) with a mean age of 59.27 years (SD = 13.77), ALS with behavioural impairment (ALSbi) with a mean age of 62.00 years (SD = 10.87), ALS with cognitive and behavioural impairment (ALScbi) with a mean age of 61.63 years (SD = 12.18), and ALS with Frontotemporal Dementia (ALS-FTD) with a mean age of 67.25 years (SD = 7.63). Of the participants, 29% had possible ALS (n = 24), 28% had probable ALS (n = 23), 17% had definite ALS (n = 14), and 26% were not classifiable by the El Escorial criteria due to presenting a pure upper or lower motor neuron syndrome (n = 22).	Regarding self-ratings, statistically relevant subclinical increases in behavioural abnormality were self-reported by all patient groups across the apathy and total behavioural change domains. The ALSni patients further self-reported increased disinhibition, while the ALScbi patients self-reported increased disinhibition as well as executive dysfunctioning and the ALS-FTD patients self-reported increased executive dysfunction. Apathy: There was strong evidence that ALSbi patients retained less insight into apathy than ALSni patients and moderate evidence compared to ALSci patients. Evidence regarding the expected group differences between the ALS-FTD/-cbi patients and ALSni/-ci patients was inconclusive. Disinhibition: ALS-FTD patients retained a worse insight into disinhibition than ALSci patients. There was very strong evidence that ALSbi patients retained a worse insight into disinhibition than ALSni patients and strong evidence compared to ALSci patients. Evidence regarding the expected group differences between the ALScbi patients and ALSni/-ci patients was inconclusive. Clinically, only ALS-FTD patients lost insight into their increasing disinhibition. Executive dysfunction: ALSbi patients retained a worse insight into their executive dysfunction than ALSni patients and moderate insight compared to ALSci patients. Evidence regarding the expected group differences between the ALS-FTD/-cbi patients and ALSni/-ci patients was inconclusive. Total Behavioural Issues: None of the Strong profile groups lost insight into their overall behavioural decline.
Tipton et al. [100]	*C9orf72seq* (n = 88) *MAPT* (n = 53) *GRN* (n = 43).	Participants were screened through Advancing Research and Treatment in Frontotemporal Lobar Degeneration (ARTFL), Longitudinal Evaluation of Familial Frontotemporal Dementia Subjects (LEFFTDS), and the ARTFL LEFFTDS Longitudinal Frontotemporal Lobar Degeneration Consortium (ALLFTD), involving 14 study centres. These individuals had a single pathogenic variant in the *C9orf72*, *MAPT*, or *GRN* genes. The participants were between 22 and 85 years of age at the time of evaluation, with no structural brain lesions or other known neurological disorders. A total of 184 symptomatic participants carried a single pathogenic variant in *C9orf72*, *MAPT*, or *GRN*.	Behavioural Symptoms and Motor Symptoms: Patients with *C9orf72seq* mutations typically experience the onset of behavioural symptoms at a median age of 56 years, earlier than the *GRN* group (median of 61 years) and similar to *MAPT* patients (median of 50 years). Motor symptoms onset for *C9orf72seq* is earlier (median of 59 years) compared to *GRN* (median of 64 years) and *MAPT* (median of 49 years). Predominant Domain of Initial Change: The initial domain of change varies across groups. Cognitive changes are noted first in 26.1% of *C9orf72seq* patients, while 55.6% of *GRN* patients initially present with behavioural symptoms. *MAPT* patients also predominantly show behavioural symptoms first (66.0%).
Tondo et al. [101]	*C9orf72seq* (n = 6) *SOD1* (n = 1) *TBK1* (n = 1) *KIF5A* (n = 1)	The study cohort included ten ALS patients referred to the AL Tertiary Centre at the “Maggiore della Carit` a” University Hospital (Novara, Italy) from January 2018 to February 2022. All patients had symptomatic FTD. *C9orf72seq*: Age between 45 and 65. *SOD*: Age between 40 and 50. *TBK*: Age between 60 and 65. *KIF5A*: Age between 60 and 65. No differences between genders were provided.	In the *C9orf72seq* group, patients were divided into fast progressors (monthly decrease in ALSFRS-R score from symptoms’ onset to 18F-FDG-PET scan > 0.9/month) or slow progressors (monthly decrease in ALSFRS-R score from symptoms’ onset to 18F-FDG-PET scan > 0.9/month). The fast progressors showed moderate to severe cognitive impairment, involving mainly executive functions and language. Patients considered as slow progressors had no consistent cognitive impairment. The patients with *SOD1*-ALS had a normal cognitive status, without neuropsychological deficits. The baseline neurocognitive evaluation of the *TBK1* mutation showed intact cognitive functioning but with a worsening in long-term memory deficit and executive functions over the disease’s course. The patient carrying the *KIF5A* mutation showed a primary involvement of working memory (Digit Span Test) and a mild involvement of the executive functions, classifying the patient as ALS-ci following the Strong criteria.
Van Deerlin et al. [102]	*GRN* (n = 9)	All patients had FTLD. *GRN*: Mean age of 55 years (SD = 10.4). The group comprised 55.6% females.	In patients with GRN mutations, cognitive impairments were primarily observed in recognition memory (word-list learning and memory recall), where these individuals showed significant difficulty compared to matched controls. However, these patients exhibited relatively modest language deficits, which were less pronounced than in patients with FTLD-U who did not have a GRN mutation. Specifically, some individual cases with GRN mutations showed minor deficits in verbally mediated tasks. In contrast, patients without the GRN mutation displayed significant language difficulties, particularly in tasks related to confrontation naming and category naming fluency, assessed using the Boston Naming Test and verbal fluency tasks (e.g., category fluency for “animals”)
Van Langenhove et al. [11]	*MAPT* (n = 8), *GRN* (n = 27), *C9orf72seq* (n = 26)	All patients have symptomatic FTLD. *C9orf72seq*: Mean age of 55.3 years (SD = 7.8). The group comprises 83% females. *GRN*: Mean age of 59.6 years (SD = 7.3). The group comprises 89% females. *MAPT*: Mean age of 56.9 years (SD = 4.4). The group comprises 95% females.	Memory: Reported by 42% of the patients. However, it did not resemble the early isolated episodic memory disorder typically seen in Alzheimer’s disease. Language: Patients with C9orf72seq mutations showed varied language impairments: some had primary non-fluent aphasia (PNFA) with non-fluent speech and agrammatism, while others had semantic dementia (SD) with severe anomia and comprehension problems. Some developed overlapping symptoms of bvFTD over time. Behavioural Assessment: In 85% of *C9orf72seq* expansion carriers diagnosed with bvFTD, key symptoms included disinhibited and socially inappropriate behaviour, restlessness, and hyperactivity. Apathy without disinhibition was rare (14%). Compulsive behaviour was common, and 12% had psychotic features.
Vinceti et al. [103]	*C9orf72seq* (n = 1)	A 54-year-old woman showing cognitive impairment and behavioural disturbances with both neuroimaging and cerebrospinal fluid (CSF) biomarkers consistent with AD pathology, her 49-year-old brother with typical FTD-ALS, and their 63-year-old mother with the behavioural variant of FTD and CSF biomarkers suggestive of AD pathology.	At Time A (onset, during hospitalisation), the patient’s language abilities were somewhat impaired, as indicated by scores on the Boston Naming Test and their semantic fluency being below normal values. Visuospatial processing was not assessed. Short-term memory was also compromised, with a Digit Span of 5 and Corsi Span of 3, both falling short of typical performance standards. Anterograde memory was particularly weak, with very low scores on the Babcock story, verbal paired-associate learning, and the Free and Cued Selective Reminding Test (FCSRT). Executive functions were also notably impaired, as evidenced by low scores on the Cancellation Test, phonemic fluency, and high completion times and errors on the Stroop Test and Trail Making Test (TMT). By Time B (two months after discharge), there were improvements in some areas, such as increased scores in semantic fluency and phonemic fluency, though still below normal values. The patient’s visuospatial processing, as measured by the Rey–Osterrieth Complex Figure (ROCF) copy, was still slightly below normal. Short-term memory and anterograde memory showed some improvement but remained below expected levels, with particularly low scores in the FCSRT and delayed recall tasks. Executive function improvements were seen in the Cancellation Test and the Frontal Assessment Battery (FAB), yet performance on TMT and Stroop tasks continued to be poor, indicating ongoing difficulties in executive functioning.
Wang et al. [104]	*ANXA11* (n = 1)	Ten probands/patients with suspected ALS–FTD or FTD from the Department of Neurology, China–Japan Friendship Hospital in Beijing, were enrolled in the study from July 2019 to January 2022. In total, six probands presented with ALS–FTD, and four with behaviour variant FTD(bvFTD).A non-synonymous heterozygous mutation (c. A>G, p.D G) of ANXA11 in proband1 was found, which is associated with ALS. All 10 patients (6 men and 4 women) diagnosed with bvFTD were from the Chinese mainland. The onset of symptoms occurred at the age of 34–80 years; median (IQR) was 69 (58.5–73.5). All 10 patients showed behavioural and executive deficits and anomia. There were six patients with positive family histories.	The cognitive features and clinical characteristics of the patients, listed by proband number, exhibit a range of findings across different ages, disease durations, and cognitive measures. The ages of onset for the patients span from 34 to 80 years, with a disease duration ranging from 8 to 36 months. The gender distribution includes both males and females, with varying educational backgrounds from 2 to 16 years. Cognitive and behavioural symptoms are consistently noted, with many patients exhibiting deficits in executive functions and anomia. The Mini-Mental State Examination (MMSE) scores range from 19 to 27, indicating varying levels of cognitive impairment. The Montreal Cognitive Assessment (MoCA) scores also vary, from 18 to 25. Other cognitive measures, such as the Digit Span Test (DST) for forwards and backwards, show consistent scores across patients, with no significant deviations. Verbal Fluency Tests (VFTs) range widely, from 21 to 50, indicating significant variability in language function. The Trail Making Test (TMT-B) completion times range from 50 to 244 s, reflecting differences in executive processing speed. Memory assessments, including the Rey Auditory Verbal Learning Test (RAVLT) and the Boston Naming Test (BNT), show scores from 29 to 42 and 18 to 25, respectively, suggesting diverse memory capabilities among patients. Lastly, the Stroop Colour and Word Test (CWT) scores vary from 29 to 40, highlighting differences in cognitive control and processing speed.
Wicks et al. [105]	*SOD1* (n = 7), Control (n = 35)	*SOD1* FALS (Familial Amyotrophic Lateral Sclerosis with *SOD1* Mutation): Mean age of 48.57 years (SD = 9.7). The group comprises 57.1% females. Controls: Mean age of 51.86 years (SD = 10.5). The group comprises 37.8% females.	Memory: There were no notable differences in memory performance between *SOD1* FALS patients and controls. Language: *SOD1* FALS patients showed a comparable performance to controls in tasks assessing executive functions, including verbal fluency (WVFi). In the Graded Naming Test, *SOD1* FALS patients performed comparably to controls. They did not show significant differences in action naming or noun naming compared to controls. Executive Function: Tested using the Hayling Sentence Completion Test, SOD1 FALS patients scored similarly to controls. Behavioural Measures: *SOD1* FALS patients had higher levels of apathy compared to controls, but this was likely due to the physical limitations of the disease rather than cognitive decline. There were no significant differences in other behavioural measures, such as frontal behaviour changes. Emotional Lability: Both *SOD1* FALS and SALS patients exhibited higher levels of emotional lability compared to controls, indicating that emotional changes can occur independently of cognitive impairment in ALS.
Wiesenfarth et al. [106]	*C9orf72seq* (n = 248) *SOD1* (n = 84) Sporadic patients without genetic mutation (n = 2178)	Patients were enrolled from the MND-net database. A total of 248 patients with amyotrophic lateral sclerosis (ALS) carrying *C9orf72* mutations were studied. Comparator groups included 84 patients with *SOD1* mutations and 2178 sporadic ALS patients without evidence of a causative genetic mutation, all of whom were similarly enrolled from the MND-net database, analogous to the patients carrying *C9orf72* mutations.	The neuropsychological characteristics of patients assessed with the Edinburgh Cognitive and Behavioural Screen (ECAS) across three groups—*C9orf72seq* mutation carriers, sporadic ALS patients, and SOD1 mutation carriers. Non-ALS-Specific Cognitive Scores: No significant differences were observed between the groups in non-ALS-specific cognitive performance. Memory: There were no significant differences in memory performance across the groups. Spatial Perception: Spatial perception scores were similar across all groups, with no significant differences observed. ALS-Specific Cognitive Scores: While there was a trend towards better performance in *SOD1* mutation carriers compared to sporadic ALS and *C9orf72seq* carriers, the difference was only statistically significant between *SOD1* carriers and sporadic ALS patients. Verbal Fluency: *SOD1* mutation carriers showed a significantly better verbal fluency compared to both sporadic ALS patients and *C9orf72seq* carriers. Language: No significant differences in language performance were found between the groups. Executive Functions: *SOD1* mutation carriers demonstrated a significantly better executive function compared to sporadic ALS patients, while no significant differences were observed between *C9orf72seq* carriers and sporadic ALS patients. Total ECAS Score: SOD1 mutation carriers had significantly higher total cognitive scores compared to sporadic ALS patients, while the difference between C9orf72seq carriers and sporadic ALS patients was not statistically significant.
Wilke et al. [107]	*C9orf72seq* (n = 117) *GRN* (n = 104) *MAPT* (n = 49) Controls (n = 174)	All subjects from the GENFI cohort were included if they had at least one available serum sample (n = 444; sample collection: 2015–2019). The presymptomatic group had a balanced gender distribution, with 37% male participants and a median age of 41.2 years. Among C9orf72 mutation carriers (median follow-up duration: 2.15 years), 36% were male, and their median age was 42.5 years. GRN mutation carriers had a median follow-up duration of 1.10 years, with 39% male and a median age of 41.2 years. MAPT mutation carriers had a median follow-up duration of 1.98 years, with 35% male participants and a median age of 36 years. The converters group consisted of 7 individuals, with a median follow-up duration of 1.68 years. This group had a higher proportion of males (71%) and a median age of 62.5 years. The symptomatic cohort included 91 individuals, with a median follow-up duration of 1.13 years. This group had a median age of 63.3 years and 58% male participants. Among the 47 symptomatic individuals with the *C9orf72sep* mutation, the median follow-up duration was 1.22 years, with a median age of 64.6 years and 66% male. *GRN* mutation carriers had a median follow-up duration of 1.02 years, a median age of 63.4 years, and 48% male. *MAPT* mutation carriers had a median follow-up duration of 2.04 years, with a median age of 62.5 years and 53% male. The non-carriers group had a median follow-up duration of 1.18 years. This group had a median age of 44.1 years and 43% male participants.	Presymptomatic Mutation Carriers: Cognitive assessment scores at baseline indicated no cognitive impairment, with a median MMSE score of 30 and a CDR plus NACC-FTLD score of 0. *C9orf72seq* Mutation Carriers: At baseline, their cognitive performance was well-preserved, as reflected by a median MMSE score of 30 and a CDR plus NACC-FTLD score of 0. *GRN* Mutation Carriers: Cognitive function at baseline was intact, with a median MMSE score of 30 and a CDR plus NACC-FTLD score of 0, indicating no observable cognitive deficits. *MAPT* Mutation Carriers: Baseline cognitive assessments showed no significant impairment, with a median MMSE score of 30 and a CDR plus NACC-FTLD score of 0. Converters (Transition to Symptomatic Stage): Cognitive performance before conversion showed a median MMSE score of 30 and a CDR plus NACC-FTLD score of 0, indicating no cognitive impairment at baseline. The median age at conversion to symptomatic status was 65.7 years, illustrating the progression from a presymptomatic to symptomatic stage over the follow-up period. Symptomatic Individuals: At baseline, cognitive impairment was evident, with a median MMSE score of 25 and a CDR plus NACC-FTLD score of 2, reflecting moderate cognitive decline. The median disease duration at assessment was 4.4 years, indicating a substantial period of symptomatic progression. *C9orf72seq*-Symptomatic: Cognitive function at baseline was impaired, with a median MMSE score of 26 and a CDR plus NACC-FTLD score of 2. The median disease duration was 5.4 years, suggesting a progressive decline in cognitive function over time. *GRN*-Symptomatic: Cognitive assessments revealed significant impairment, with a median MMSE score of 22 and a CDR plus NACC-FTLD score of 2. The median disease duration was 2.7 years, indicating the ongoing progression of cognitive symptoms. *MAPT*-Symptomatic: At baseline, they exhibited cognitive impairment, with a median MMSE score of 24 and a CDR plus NACC-FTLD score of 2. The median disease duration was 6.0 years, reflecting a longer period of symptomatic progression compared to other mutation carriers. Non-Carriers: Cognitive function was preserved at baseline, with a median MMSE score of 30 and a CDR plus NACC-FTLD score of 0, indicating no cognitive impairment. This group serves as a comparison cohort to assess the impact of genetic mutations on cognitive function.
Wood et al. [108]	R406W *MAPT* (n = 2)	Case 1 was a 66-year-old woman with 12 years of worsening personality changes and ritualistic behaviours. Case 2 was a 64-year-old woman with 13 years of behavioural changes, including confabulation and a preference for sweet foods.	Global Cognition: Both patients showed progressive cognitive decline. For Case 1, the MMSE revealed scores of 13/30 and 16/30 across different years. Case 2’s MMSE scores were 28/30, 25/30, and 25/30 in various assessments, indicating a decline in cognitive function over time. Language: Language abilities were compromised. Case 1 showed fluent but content-poor speech and surface dyslexia. Neuropsychological testing included confrontational naming and lexical fluency assessments, showing significant impairment. Case 2 exhibited difficulties in naming and verbal memory with tests such as the Graded Naming Test and confrontation naming tasks. Memory: Memory testing revealed significant impairments. Case 1 had deficits in episodic memory, including poor performance on the Rey–Osterrieth Complex Figure and the Camden Short Recognition Memory Test for Words (CSRMT-W). Case 2 demonstrated severe impairments in episodic memory with low scores on tests like the Short Story—Delayed Recall and the CSRMT-W. Attention and Executive Functioning: Tests assessing executive functions revealed deficits. For Case 1, assessments like the WAIS-III and verbal fluency tasks indicated a frontal dysexecutive syndrome. Case 2’s performance on the WAIS-III and the lexical and semantic fluency tests also reflected executive dysfunction. Behaviour and Emotion: Behavioural changes were notable, with both patients displaying altered eating habits and social behaviours. Case 1 showed ritualistic behaviours and apathy, while Case 2 exhibited confabulation and impulsive behaviours. These changes were observed through clinical assessments and behavioural observations.
Yang et al. [109]	*C9orf72seq* (n = 1) *ANXA11* (n = 2) *CCNF* (n = 1) *UBLQN2* (n = 1)	A total of 1208 patients, including 66 familial ALS (FALS) and 1142 sporadic ALS (SALS) patients, were included. Twenty-three patients with sporadic ALS and one familial ALS index had concomitant FTD, which accounted for 1.99% (24/1208) of patients with ALS. In sporadic ALS-FTD, one case harbouring the *C9ORF72SEQ* expansion variant, two cases harbouring *ANXA11* variants, and one individual carrying the *CCNF* variant were identified. A recurrent *UBQLN2* variant was detected in a familial ALS-FTD patient. The total cohort included a majority who were male (58.4%). The average age at onset was 49.3 years, with a standard deviation of 11.4 years. Among these patients, 14.1% presented with bulbar onset, and 5.5% had a family history of amyotrophic lateral sclerosis (ALS). Within the 24 patients diagnosed with ALS-FTD, 62.5% were male.	*UBQLN2* Variant: The patient with the *UBQLN2* p.P500S variant, who had bulbar onset ALS, showed a marked decline in cognitive function as indicated by her Mini-Mental State Examination (MMSE) score of 21/30 and a Montreal Cognitive Assessment (MoCA) score of 18/30. This cognitive impairment, along with significant motor symptoms and eventual respiratory failure, occurred over a 24-month period. The lack of detailed cognitive assessment data for her family members, including her brother who also had ALS, limits the understanding of familial cognitive patterns *C9ORF72SEQ* Repeat Expansion Mutation: The individual with the *C9ORF72SEQ* repeat expansion experienced limb onset ALS and exhibited notable cognitive deficits, with an MMSE score of 22/30 and a MoCA score of 17/30. This patient also displayed behavioural changes, such as a loss of empathy and repetitive actions, and succumbed to respiratory failure after 24 months of disease duration. The cognitive decline observed is consistent with the known effects of the C9ORF72SEQ mutation. *ANXA11* Variant: The carrier of the *ANXA11* p.P36R variant presented with ALS and behavioural variant frontotemporal dementia (bvFTD). His cognitive assessments revealed a significant impairment, with an MMSE score of 20/30 and a MoCA score of 15/30. The patient exhibited behavioural changes such as irritability and inappropriate behaviour, alongside neuroimaging findings of frontotemporal atrophy and hypometabolism. Another patient with this variant also showed cognitive impairment, although specific details of cognitive tests were not provided. *CCNF* Variant: The patient with the *CCNF* p.V167M variant, who developed upper limb weakness and bvFTD, had cognitive assessments showing an MMSE score of 23/30 and a MoCA score of 19/30. This indicates moderate cognitive impairment in conjunction with his motor symptoms and behavioural issues, leading to death from respiratory failure after 11 months.

Abbreviations: ACE, Addenbrooke’s Cognitive Examination; ALS, amyotrophic lateral sclerosis; BNT, Boston Naming Test; *C9orf72*, chromosome 9 open reading frame 72; CSRMT-W, Camden Short Recognition Memory Test for Words; FAB, Frontal Assessment Battery, FTD, frontotemporal dementia; *GRN*, granulin; *MAPT*, microtubule-associated protein tau; MINT, Multilingual Naming Test; MMSE; Mini-Mental State Examination; MND, motor neuron disease; MoCA, Montreal Cognitive Assessment; PALPA, Psycholinguistic Assessment of Language Processing in Aphasia; PNFA, primary non-fluent aphasia; PPA, primary progressive aphasia; RAVLT, Rey Auditory Verbal Learning Test; RBMT, Rivermead Behavioural Memory Test; SCWT I and II, Stroop Colour and Word Test; VAT, Visual Association Test; WCST, Wisconsin Card Sorting Test; WVFi, Verbal Fluency Index.

## Data Availability

The data that support the findings of this study are available from the corresponding author upon reasonable request.

## Abbreviations

The following abbreviations are used in this manuscript:

ACE-III	Addenbrooke’s Cognitive Examination III
ACE-R	Addenbrooke’s Cognitive Examination—Revised
ALS	Amyotrophic Lateral Sclerosis
*ANXA11*	*Annexin A11*
BNT	Boston Naming Test
bvFTD	Behavioural variant Frontotemporal Dementia
*C9orf72seq*	*Chromosome 9 Open Reading Frame 72*
CBI	Cambridge Behavioural Inventory
CBI-R	Cambridge Behavioural Inventory Revised
*CCNF*	*Cyclin F*
CDR	Clinical Dementia Rating
CDR-FTD	Clinical Dementia Rating–Frontotemporal Dementia version
*CHMP2B*	*Charged Multivesicular Body Protein 2B*
CVLT-SF	California Verbal Learning Test–Short Form
*DCTN1*	*Dynactin 1*
DS	Digit Span
ECAS	Edinburgh Cognitive and Behavioural ALS Screen
FAB	Frontal Assessment Battery
FALS	Familial Amyotrophic Lateral Sclerosis
FDG-PET	Fluorodeoxyglucose Positron Emission Tomography
FBI	Frontal Behavioural Inventory
FRS	Frontotemporal Dementia Rating Scale
FTD	Frontotemporal Dementia
FTLD	Frontotemporal Lobar Degeneration
*FUS*	*Fused in Sarcoma*
*GRN*	*Progranulin gene mutation*
HADS	Hospital Anxiety and Depression Scale
*HNRNPA1*	*Heterogeneous Nuclear Ribonucleoprotein A1*
MAPT	Microtubule Associated Protein Tau
MINT	Multilingual Naming Test
MMSE	Mini-Mental State Examination
MoCA	Montreal Cognitive Assessment
NPI	Neuropsychiatric Inventory
*PGRN*	Progranulin
PPA	Primary Progressive Aphasia
*PSEN1*	Presenilin 1
*PSEN2*	Presenilin 2
RMT	Recognition Memory Test
SALS	Sporadic Amyotrophic Lateral Sclerosis
*SOD1*	Superoxide Dismutase 1
*SQSTM1*	Sequestosome 1
STAI	State–Trait Anxiety Inventory
*TBK1*	*TANK-Binding Kinase 1*
*TARDBP*	*Transactive Response DNA Binding Protein*
TMT	Trail Making Test
*TREM2*	*Triggering Receptor Expressed on Myeloid Cells 2*
*UBQLN2*	*Ubiquilin 2*
*VCP*	*Valosin-Containing Protein*
